# Comparative proteomic analysis provides novel insight into the interaction between resistant *vs* susceptible tomato cultivars and TYLCV infection

**DOI:** 10.1186/s12870-016-0819-z

**Published:** 2016-07-19

**Authors:** Ying Huang, Hong-Yu Ma, Wei Huang, Feng Wang, Zhi-Sheng Xu, Ai-Sheng Xiong

**Affiliations:** State Key Laboratory of Crop Genetics and Germplasm Enhancement, College of Horticulture, Nanjing Agricultural University, 1 Weigang, Nanjing, 210095 Jiangsu China; College of Plant Protection, Nanjing Agricultural University, Nanjing, 210095 Jiangsu China

**Keywords:** TYLCV, Proteomic approach, 2-DE, Expression pattern, Tomato

## Abstract

**Background:**

Tomato yellow leaf curl virus (TYLCV) is a member of the family Geminiviridae, genus *Begomovirus*. The virus is a widespread plant virus that causes important economic losses in tomatoes. Genetic engineering strategies have increasingly been adopted to improve the resistance of tomatoes to TYLCV.

**Results:**

In this study, a proteomic approach was used to investigate the molecular mechanisms involved in tomato leaf defense against TYLCV infection. Proteins extracted from leaves of resistant tomato cultivar ‘Zheza-301’ and susceptible cultivar ‘Jinpeng-1’ after TYLCV infection were analyzed using two-dimensional gel electrophoresis. Eighty-six differentially expressed proteins were identified and classified into seven groups based on their functions. For several of the proteins, including CDC48, CHI and HSC70, expression patterns measured using quantitative real-time PCR differed from the results of the proteomic analysis. A putative interaction network between tomato leaves and TYLCV infection provides us with important information about the cellular activities that are involved in the response to TYLCV infection.

**Conclusions:**

We conducted a comparative proteomic study of TYLCV infection in resistant and susceptible tomato cultivars. The proteins identified in our work show a variety of functions and expression patterns in the process of tomato–TYLCV interaction, and these results contribute to our understanding of the mechanism underlying TYLCV resistance in tomatoes at the protein level.

**Electronic supplementary material:**

The online version of this article (doi:10.1186/s12870-016-0819-z) contains supplementary material, which is available to authorized users.

## Background

Tomato yellow leaf curl virus (TYLCV), is a member of the genus *Begomovirus* of the family Geminiviridae, which has three other genera, namely, *Mastrevirus*, *Curtovirus* and *Topocuvirus*, and contains a circular single-stranded DNA (ssDNA) molecule 2.7–2.8 kb in length [1, 2]. The TYLCV genome encodes six open reading frames (ORFs): two overlapping ORFs AV1 (encoding capsid protein), AV2 (encoding movement protein MP), and ORFs AC1-AC4 (encoding replication-associated protein Rep, transcriptional activator TrA, replication enhancer protein REn and induction of plant cell division protein, respectively) on the complementary-sense strand [3–8]. TYLCV, which is transmitted by the whitefly *Bemisia tabaci*, threatens up to 20 different plant species, including pumpkin, tobacco and tomato [9, 10]. TYLCV infection begins when the viruliferous whitefly *B. tabaci* feeds by inserting its proboscis into leaves, transmitting the virus to the host cells of the plant [11]. Upon entering the host cell, the ssDNA genome of TYLCV begins replicating through a rolling circle mechanism. TYLCV can spread to adjacent cells via the complex cytoplasm and plasmodesmata, inducing a new infection process.

After about 2 weeks of TYLCV infection, symptoms become increasingly apparent, with yellow and shriveled leaves, dwarfed plants, withered flowers and growth retardation, causing serious economic loss. Increasing numbers of genes involved in the response to TYLCV infection have been identified, such as *GroEL* [12], a developmentally regulated lipocalin-like gene *SlVRSLip* [13], and transcription factors, including bHLH [14], AP2/ERF [15], NAC [16]. Comparative transcriptomics and metabolomics have also been conducted in resistant and susceptible tomato cultivars to analyze the mechanisms of resistance to TYLCV infection [16, 17].

In recent years, several strategies have demonstrated the advantages of the proteomics approach for studying underlying plant physiological processes, such as plant defense response. A proteomic analysis of root proteins in avocado infected with *Phytophthora cinnamomi* identified 21 differentially expressed proteins, including homologs to glutathione S-transferase, isoflavone reductase and several abscisic acid stress-ripening proteins [18]. Moreover, a study of interaction with *Lasiodiplodia theobromae* infection in cashew plants identified 73 proteins with significantly different expression levels, which were mainly involved in energy metabolism pathways, stress and defense, protein metabolism and cell signaling [19].

Whitefly-transmitted geminiviruses such as the begomovirus TYLCV pose a serious threat to tomato production. Breeding of tomatoes resistant to TYLCV started in the late 1960s [20]. Tomato genotypes with resistance to begomoviruses are derived from different wild species. To date, five loci linked to resistance have been identified: Ty-1/Ty-3 and Ty-4 from *S. chilense*, Ty-2 from *S. habrochaites* and Ty-5 from *S. peruvianum* [17]. And two genes associated with these Ty loci, Ty-1/Ty-3 and Ty-5, have been identified [21, 22]. To investigate the tomato–TYLCV interaction, we conducted a proteomic analysis in resistant and susceptible tomato cultivars after TYLCV infection. Leaf proteins of resistant tomato cultivar ‘Zheza-301’ and susceptible cultivar ‘Jinpeng-1’ were extracted at 19 days post infection (dpi) and analyzed using two-dimensional gel electrophoresis (2-DE). Eighty-six proteins were identified and classified into different functional categories. The expression levels of 19 genes encoding these proteins were analyzed at six time points after infection (2, 4, 6, 10, 15 and 19 dpi) by quantitative real-time PCR (qRT-PCR). A putative TYLCV infection response network in tomato leaves was constructed.

This work mainly (1) identified proteins significantly expressed in response to TYLCV infection in tomatoes; (2) analyzed the proteins that were differentially expressed between resistant and susceptible tomato cultivars; (3) revealed the defense mechanism between tomato and TYLCV in proteins and biochemical process level; and (4) analyzed the interaction network in the cells of tomato leaves after TYLCV infection. The overall results contribute significantly to our understanding of protein response and alteration and provide insights into the molecular mechanisms involved in response to TYLCV infection in tomatoes.

## Results

### Symptoms of resistant and susceptible tomato cultivars in response to TYLCV infection

**Fig. 1:**
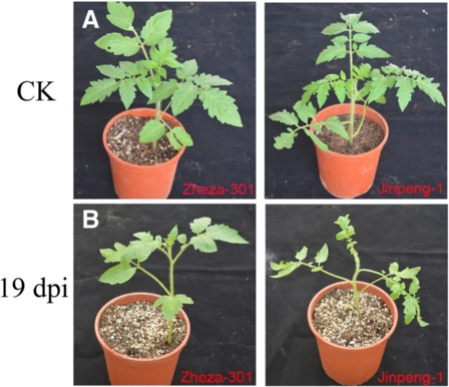
Symptoms analysis in two tomato cultivars after tomato yellow leaf. curly virus (TYLCV) infection. The figure shows typical phenotypes observed at 19 dpi in ‘Jinpeng-1’ not in ‘Zheza-301’. **a** Control tomato plants grown in normal environment. **b** Two tomato cultivars infected TYLCV at 19 dpi

**Fig. 2:**
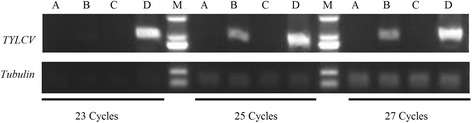
Detection of the accumulation of TYLCV CP in two tomato cultivars by semi-quantitative PCR at different PCR cycles. A: Control plant: Zheza-301. B: Treatment plant: Zheza-301 after TYLCV infection at 19 dpi. C: Control plant: Jinpeng-1. D: Treatment plant: Jinpeng-1 after TYLCV infection at 19 dpi. M: Marker

Two leaf-stage tomato cultivars (resistant variety: ‘Zheza-301’, susceptible variety: ‘Jinpeng-1’) were exposed to viruliferous whiteflies. The two tomato cultivars showed no symptoms at 10 dpi. At 17 dpi, there was a little yellowing in the leaves of ‘Jinpeng-1’ (Additional file 1: Figure S1). Compared with the control plants, ‘Jinpeng-1’ samples presented a typical TYLCV phenotype, with curly yellow leaves at 19 dpi. In contrast, ‘Zheza-301’ exhibited no symptoms (Fig. 1). For better comparative understanding of the development of TYLCV infection, the leaves of the two tomato cultivars at 19 dpi and control plants were collected and frozen in liquid nitrogen to extract protein and total genomic DNA. To detect TYLCV accumulation, semi-quantitative PCR was conducted using the primers TYLCV-01 (Additional file 2: Table S1). As shown in Fig. 2, in control plants, there was no TYLCV accumulation in either ‘Zheza-301’ or ‘Jinpeng-1’ at different PCR cycles. In TYLCV infected plants, after 23 PCR cycles, there was high expression of TYLCV DNA in ‘Jinpeng-1’, while there was weak or no expression in ‘Zheza-301’. TYLCV DNA was clearly present in ‘Zheza-301’ after about 25 cycles, but at lower levels than in ‘Jinpeng-1’.

### 2-DE analysis of tomato leaf proteins after TYLCV infection

As described above, samples for protein extraction were collected at 19 dpi, when the leaves of susceptible cultivar ‘Jinpeng-1’were yellow and curly. Total proteins of the two tomato cultivars (‘Zheza-301’: control and TYLCV infection; ‘Jinpeng-1’: control and TYLCV infection) were separated using 2-DE. Representative maps of three biological replicates are shown in Additional file 3: Figure S2.

**Fig. 3:**
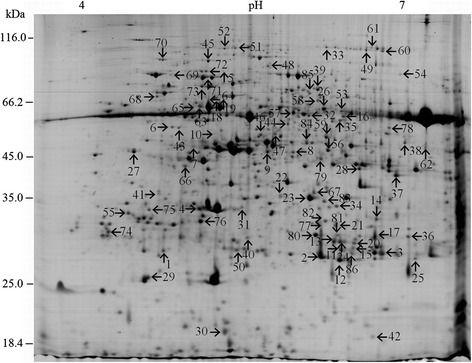
Identification of 86 leaf protein spots from two tomato cultivars. The numbers with arrows indicated the differentially expressed and identified protein spots

**Fig. 4:**
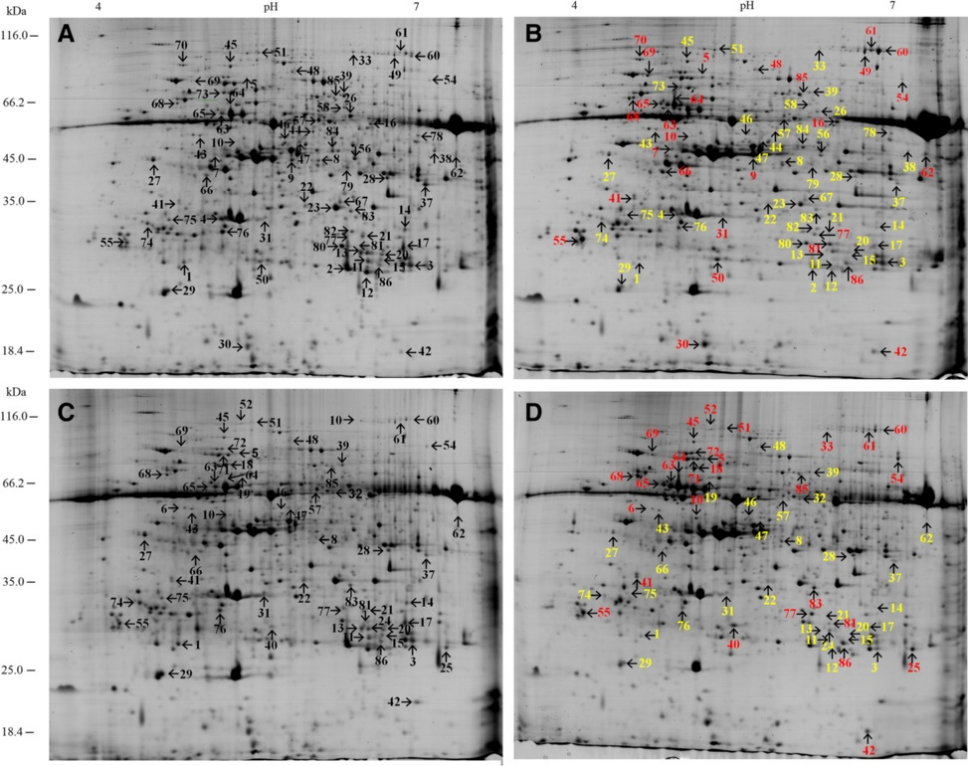
Protein profiles of identified proteins in two tomato cultivars analyzed by 2-DE. Spots in red and yellow indicate increased or decreased abundance of each sample, respectively. **a** ‘Zheza-301’: no TYLCV-infected. **b** ‘Zheza-301’: TYLCV-infected at 19 dpi. **c** ‘Jinpeng-1’: no TYLCV-infected. **d** ‘Jinpeng-1’: TYLCV-infected at 19 dpi

Over 500 protein spots were detected in each gel with different expression abundance. To detect differentially expressed proteins, the protein abundance ratios Z_T_/Z_C_ (treatment /control plants of ‘Zheza-301’) and J_T_/J_C_ (treatment/control plants of ‘Jinpeng-1’) were calculated for each spot. Z_C_/J_C_ and Z_T_/J_T_ were also calculated to analyze differences between the two tomato cultivars. A total of 86 spots with apparent molecular mass between 26.08 and 114.02 kDa and isoelectric point (*pI*) between 4.69 and 8.96 showed significantly differential expression with more than 2.0-fold or less than 0.5-fold differences in abundance ratios (Fig. 3). Identified proteins are shown in Table 1. Among the 86 protein spots, four spots (spot 34, spot 35, spot 36 and spot 53) showed no significant difference in expression with infection but significant differences in expression between the two tomato cultivars. These proteins did not respond significantly to TYLCV infection at 19 dpi, but were related to differences between the two tomato cultivars. The significantly differentially expressed proteins in TYLCV infection in the two tomato cultivars are shown in Fig. 4.

**Table 1:** A total of 86 differentially expressed proteins identified in resistant and susceptible tomato cultivars after TYLCV infection

^a^Spot no.	^b^Accession no.	^c^Protein name, Orgnaism	^d^Protein score	^e^SC	^f^MP	^g^MW (kDa) /pI	^h^Z_C_/J_C_	^h^Z_T_/J_T_	^h^Z_T_/Z_C_	^h^J_T_/J_C_
Photosynthesis										
1	gi|407970998	Chlorophyll a-b binding protein 4, *Solanum lycopersicum*	135	13	2	28.98/5.33	0.31^*^ ± 0.15	0.45^*^ ± 0.35	0.22^*^ ± 0.13	0.18^*^ ± 0.09
2	gi|115813	Chlorophyll a-b binding protein 8, *Solanum lycopersicum*	212	15	5	29.34/8.96	1.09 ± 0.22	0.45^*^ ± 0.35	0.25^*^ ± 0.06	0.90 ± 0.33
3	gi|460405507	Chlorophyll a-b binding protein 8, *Solanum lycopersicum*	81	4	1	29.26/8.65	1.34 ± 0.25	1.78 ± 0.30	0.46^*^ ± 0.03	0.36^*^ ± 0.13
4	gi|460375240	Oxygen-evolving enhancer protein 1, *Solanum lycopersicum*	273	10	2	35.15/5.91	0.84 ± 0.07	0.18^*^ ± 0.01	0.31^*^ ± 0.04	1.49 ± 0.38
5	gi|460372520	Oxygen-evolving enhancer protein 1, *Solanum lycopersicum*	99	19	2	35.48/5.84	1.93 ± 0.19	0.18^*^ ± 0.01	2.21^*^ ± 0.44	10.57^*^ ± 0.52
6	gi|460408969	Rubisco accumulation factor 1, *Solanum lycopersicum*	119	9	3	50.46/5.10	5.36^*^ ± 0.28	1.85 ± 0.28	0.77 ± 0,29	2.18^*^ ± 0.39
7	gi|460401823	Ribulose bisphosphate carboxylase/oxygenase activase 1, *Solanum lycopersicum*	56	3	1	49.05/8.15	0.09^*^ ± 0.04	0.57 ± 0.12	6.75^*^ ± 0.18	1.07 ± 0.29
8	gi|723739979	Ribulose bisphosphate carboxylase/oxygenase activase, *Solanum lycopersicum*	285	11	3	50.97/8.76	0.88 ± 0.20	0.38^*^ ± 0.30	0.07^*^ ± 0.05	0.16^*^ ± 0.01
9	gi|1778414	Ribulose 1,5-bisphosphate carboxylase/oxygenase activase, *Oryza sativa*	126	7	2	48.06/5.85	0.39^*^ ± 0.04	0.78 ± 0.16	3.45^*^ ± 0.40	1.73 ± 0.01
10	gi|100380	Ribulose bisphosphate carboxylase activase, *Nicotiana tabacum*	103	7	1	26.08/5.01	0.05^*^ ± 0.01	0.03^*^ ± 0.02	3.25^*^ ± 0.44	5.57^*^ ± 0.43
11	gi|488453358	Ribulose 1,5-bisphosphate carboxylase, *Solanum lycopersicum*	171	6	3	48.57/6.71	1.22 ± 0.14	43.48^*^ ± 0.38	0.04^*^ ± 0.01	0.01^*^ ± 0,01
12	gi|488453358	Ribulose 1,5-bisphosphate carboxylase, *Solanum lycopersicum*	313	13	5	48.57/6.71	1.58 ± 0.11	23.71^*^ ± 0.28	0.06^*^ ± 0.01	0.02^*^ ± 0.01
13	gi|488453358	Ribulose 1,5-bisphosphate carboxylase, *Solanum lycopersicum*	62	4	2	48.57/6.71	1.37 ± 0.22	4.53^*^ ± 0.25	0.05^*^ ± 0.01	0.05^*^ ± 0.01
14	gi|488453358	Ribulose 1,5-bisphosphate carboxylase, *Solanum lycopersicum*	124	8	3	48.57/6.71	0.83 ± 0.15	1.89 ± 0.20	0.09^*^ ± 0.01	0.04^*^ ± 0.01
15	gi|1293000	Ribulose 1,5-bisphosphate carboxylase/oxygenase large subunit, *Cheirodendron trigynum*	200	6	3	52.90/6.31	0.63 ± 0.04	1.18 ± 0.20	0.10^*^ ± 0.05	0.06^*^ ± 0.01
16	gi|168282	Ribulose 1,5-bisphosphate carboxylase/oxygenase large subunit, *Lactoris fernandeziana*	281	16	4	44.16/6.33	0.19^*^ ± 0.02	0.28^*^ ± 0.02	2.60^*^ ± 0.39	1.76 ± 0.24
17	gi|21069067	Ribulose 1,5-bisphosphate carboxylase/oxygenase large subunit, *Asplenium jahandiezii*	60	4	1	39.38/7.31	1.00 ± 0.26	0.29^*^ ± 0.10	0.02^*^ ± 0.01	0.09^*^ ± 0.08
18	gi|460379814	RubisCO large subunit-binding protein subunit beta, *Solanum lycopersicum*	203	10	4	64.53/5.46	5.11^*^ ± 0.34	2.87^*^ ± 0.15	1.26 ± 0.06	2.24^*^ ± 0.19
19	gi|460366131	RubisCO large subunit-binding protein subunit beta, *Solanum lycopersicum*	53	4	2	63.24/5.72	1.15 ± 0.36	2.81^*^ ± 0.25	0.69 ± 0.08	0.28^*^ ± 0.09
20	gi|92087012	Ribulose bisphosphate carboxylase large chain OS, *Solanum lycopersicum*	65	6	3	53.43/6.55	0.77 ± 0.12	10.02^*^ ± 0.24	0.09^*^ ± 0.01	0.01^*^ ± 0.01
21	gi|92087012	Ribulose bisphosphate carboxylase large chain OS, *Solanum lycopersicum*	121	14	6	53.43/6.55	1.18 ± 0.07	32.57^*^ ± 0.19	0.08^*^ ± 0.01	0.01^*^ ± 0.01
22	gi|460375527	Ferredoxin–NADP reductase, leaf-type isozyme, *Solanum lycopersicum*	118	13	3	40.90/8.67	0.28^*^ ± 0.07	0.93 ± 0.47	0.42^*^ ± 0.10	0.14^*^ ± 0.02
23	gi|460373374	FerredoxinvNADP reductase, leaf-type isozyme, *Solanum lycopersicum*	299	20	5	40.77/8.37	0.82 ± 0.07	0.61 ± 0.07	0.48^*^ ± 0.02	0.65 ± 0.05
24	gi|350537679	Carbonic anhydrase, *Solanum lycopersicum*	278	17	4	34.85/6.67	1.45 ± 0.12	3.21^*^ ± 0.27	0.85 ± 0.12	0.38^*^ ± 0.05
25	gi|350537679	Carbonic anhydrase, *Solanum lycopersicum*	98	5	1	34.85/6.67	0.65 ± 0.15	0.35^*^ ± 0.03	1.53 ± 0.18	2.81^*^ ± 0.23
26	gi|460389468	Protein TIC 62, *Solanum lycopersicum*	313	13	5	52.96/7.66	1.02 ± 0.14	0.20^*^ ± 0.11	0.13^*^ ± 0.04	0.70 ± 0.17
27	gi|460372959	Peptidyl-prolyl cis-trans isomerase CYP38, *Solanum lycopersicum*	185	17	5	49.54/5.00	0.92 ± 0.12	1.61 ± 0.38	0.26^*^ ± 0.05	0.15^*^ ± 0.04
Carbohydrate metabolism and energy										
28	gi|469517896	Glyceraldehyde-3-phosphate dehydrogenase, *Atropa belladonna*	101	10	4	34.16/6.04	0.88 ± 0.11	0.98 ± 0.29	0.46^*^ ± 0.03	0.44^*^ ± 0.13
29	gi|460414390	ATP synthase delta chain, *Solanum lycopersicum*	166	6	4	27.32/8.90	0.49^*^ ± 0.17	1.62 ± 0.23	0.38^*^ ± 0.05	0.11^*^ ± 0.03
30	gi|303279681	Aryl-al/cohol dehydrogenase related protein, *Micromonas pusilla*	54	3	1	41.07/8.65	0.53 ± 0.11	1.05 ± 0.21	2.49^*^ ± 0.35	1.28 ± 0.19
31	gi|460397188	Thiamine thiazole synthase, *Solanum lycopersicum*	142	14	4	37.68/5.42	0.30^*^ ± 0.21	3.06^*^ ± 0.09	2.25^*^ ± 0.07	0.23^*^ ± 0.17
32	gi|460365268	Biotin carboxylase 1, *Solanum lycopersicum*	413	13	6	59.08/6.52	0.68 ± 0.27	1.51 ± 0.26	0.82 ± 0.08	0.37^*^ ± 0.13
33	gi|723747143	Aconitate hydratase, *Solanum lycopersicum*	218	5	4	107.83/6.52	67.86^*^ ± 0.19	3.13^*^ ± 0.22	0.26^*^ ± 0.02	5.77^*^ ± 0.85
34	gi|350538543	Succinic semialdehyde reductase isofom 2, *Solanum lycopersicum*	123	7	2	38.94/8.50	2.01^*^ ± 0.26	2.52^*^ ± 0.10	1.24 ± 0.09	1.06 ± 0.93
35	gi|460408278	Enolase-like, *Solanum lycopersicum*	207	11	4	48.21/5.99	3.37^*^ ± 0.29	4.38^*^ ± 0.04	1.49 ± 0.09	1.14 ± 0.14
36	gi|460415839	Probable ATP synthase 24 kDa subunit, *Solanum lycopersicum*	78	5	1	27.75/8.69	0.44^*^ ± 0.08	0.49^*^ ± 0.12	1.31 ± 0.09	1.02 ± 0.28
37	gi|460373820	ATP synthase gamma chain, *Solanum lycopersicum*	167	12	3	41.75/8.15	1.93 ± 0.33	3.80^*^ ± 0.24	0.09^*^ ± 0.01	0.05^*^ ± 0.01
38	gi|460365435	Isocitrate dehydrogenase [NADP], *Solanum lycopersicum*	172	12	4	47.00/6.35	1.05 ± 0.22	0.27^*^ ± 0.12	0.21^*^ ± 0.03	0.86 ± 0.14
39	gi|350535679	Cytosolic NADP-malic enzyme, *Solanum lycopersicum*	167	6	3	64.63/5.71	2.04^*^ ± 0.20	1.20 ± 0.14	0.48^*^ ± 0.07	0.49^*^ ± 0.20
40	gi|31088232	Chitinase, Solanum lycopersicum	123	18	7	28.05/5.93	0.82 ± 0.11	0.30^*^ ± 0.03	1.51 ± 0.31	4.12^*^ ± 0.10
Proteometabolism										
41	gi|350535160	Wound-inducible carboxypeptidase precursor, *Solanum lycopersicum*	191	5	2	56.04/5.84	1.14 ± 0.07	0.78 ± 0.05	3.62^*^ ± 0.30	5.27^*^ ± 0.22
42	gi|350535160	Wound-inducible carboxypeptidase precursor, *Solanum lycopersicum*	177	5	3	56.04/5.84	1.07 ± 0.17	0.48^*^ ± 0.02	2.72^*^ ± 0.19	6.05^*^ ± 0.30
43	gi|350534564	26S protease regulatory subunit 6A homolog, *Solanum lycopersicum*	253	17	6	47.70/4.94	1.18 ± 0.05	0.78 ± 0.25	0.20^*^ ± 0.07	0.30^*^ ± 0.04
44	gi|460393754	26S protease regulatory subunit 6B homolog, *Solanum lycopersicum*	198	7	2	46.80/5.63	1.24 ± 0.40	0.38^*^ ± 0.13	0.33^*^ ± 0.23	0.91 ± 0.34
45	gi|460400419	Elongation factor G, *Solanum lycopersicum*	258	11	5	86.79/5.45	3.75^*^ ± 0.31	0.53 ± 0.02	0.47^*^ ± 0.01	3.29^*^ ± 0.39
46	gi|460415494	Eukaryotic initiation factor 4A-2, *Solanum lycopersicum*	370	18	7	47.10/5.46	1.82 ± 0.06	1.72 ± 0.15	0.35^*^ ± 0.02	0.37^*^ ± 0.02
47	gi|460399092	Eukaryotic initiation factor 4A-2, *Solanum lycopersicum*	304	13	4	47.14/5.54	1.22 ± 0.22	2.73^*^ ± 0.07	0.49^*^ ± 0.22	0.22^*^ ± 0.05
48	gi|460391817	Elongation factor TuB, *Solanum lycopersicum*	125	5	2	56.29/6.69	0.62 ± 0.16	4.33^*^ ± 0.23	2.45^*^ ± 0.23	0.34^*^ ± 0.03
49	gi|460399098	Elongation factor 2 isoform X1, *Solanum lycopersicum*	248	7	4	94.97/5.84	1.35 ± 0.24	3.39^*^ ± 0.23	3.06^*^ ± 0.11	1.22 ± 0.19
50	gi|460396224	Eukaryotic translation initiation factor 3 subunit K, *Solanum lycopersicum*	134	11	2	26.55/5.28	0.46^*^ ± 0.03	1.12 ± 0.17	2.80^*^ ± 0.26	1.15 ± 0.12
51	gi|460391351	Cell division cycle protein 48 homolog, *Solanum lycopersicum*	172	9	5	90.15/5.10	24.39^*^ ± 0.39	1.57 ± 0.13	0.38^*^ ± 0.04	5.90^*^ ± 0.81
52	gi|460411520	Cell division cycle protein 48 homolog, *Solanum lycopersicum*	138	5	3	90.25/5.20	5.85^*^ ± 0.22	0.91 ± 0.08	1.09 ± 0.04	7.00^*^ ± 0.60
53	gi|2492530	Leucine aminopeptidase 2, *Solanum lycopersicum*	79	8	3	60.08/8.18	4.80^*^ ± 0.15	2.68^*^ ± 0.29	0.81 ± 0.07	1.46 ± 0.07
54	gi|350536267	Subtilisin-like protease precursor, *Solanum lycopersicum*	301	5	3	79.57/6.25	0.24^*^ ± 0.02	0.07^*^ ± 0.01	4.17^*^ ± 0.29	14.46^*^ ± 0.40
55	gi|460381101	28 kDa ribonucleoprotein, *Solanum lycopersicum*	100	8	3	32.69/4.70	1.11 ± 0.19	1.15 ± 0.31	2.17^*^ ± 0.70	2.05^*^ ± 0.25
Amino acid metabolism										
56	gi|723717714	Phosphoglycerate kinase, *Solanum lycopersicum*	275	17	6	42.26/5.78	1.24 ± 0.46	0.47^*^ ± 0.02	0.42^*^ ± 0.02	1.11 ± 0.41
57	gi|460404838	Adenosylhomocysteinase, *Solanum lycopersicum*	119	12	4	53.59/5.57	1.10 ± 0.34	0.53 ± 0.16	0.21^*^ ± 0.06	0.45^*^ ± 0.19
58	gi|460395681	Ketol-acid reductoisomerase, *Solanum lycopersicum*	68	5	2	64.19/6.31	0.83 ± 0.40	0.34^*^ ± 0.20	0.39^*^ ± 0.09	0.97 ± 0.21
59	gi|460415192	S-adenosylmethionine synthase 2, *Solanum lycopersicum*	207	7	2	43.51/5.41	2.09^*^ ± 0.26	2.04^*^ ± 0.07	0.64 ± 0.06	0.86 ± 0.09
60	gi|460399143	Glycine dehydrogenase (decarboxylating), *Solanum lycopersicum*	192	9	6	114.02/6.69	1.90 ± 0.14	1.39 ± 0.08	4.02^*^ ± 0.25	5.51^*^ ± 0.19
61	gi|460399143	Glycine dehydrogenase (decarboxylating), *Solanum lycopersicum*	153	7	5	114.02/6.69	3.80^*^ ± 0.19	5.08^*^ ± 0.31	6.81^*^ ± 0.38	5.09^*^ ± 0.21
62	gi|460370413	Glycine dehydrogenase, *Solanum lycopersicum*	183	9	3	42.52/6.56	0.28^*^ ± 0.02	12.57^*^ ± 0.28	3.33^*^ ± 0.16	0.07^*^ ± 0.02
63	gi|170458	Threonine deaminase, partial, *Solanum lycopersicum*	194	9	4	65.05/5.26	1.01 ± 0.01	0.82 ± 0.04	5.90^*^ ± 0.09	7.29^*^ ± 0.29
64	gi|170458	Threonine deaminase, partial, *Solanum lycopersicum*	443	18	7	65.05/5.26	0.22^*^ ± 0.01	0.40^*^ ± 0.02	5.28^*^ ± 0.62	2.96^*^ ± 0.28
65	gi|170458	Threonine deaminase, partial, *Solanum lycopersicum*	175	7	3	65.05/5.26	0.97 ± 0.01	0.55 ± 0.02	4.35^*^ ± 0.03	7.62^*^ ± 0.14
66	gi|460398434	Cysteine synthase, *Solanum lycopersicum*	317	20	4	41.26/5.41	0.99 ± 0.02	6.39^*^ ± 0.21	2.11^*^ ± 0.22	0.33^*^ ± 0.04
67	gi|460404180	Cysteine synthase, *Solanum lycopersicum*	274	17	5	34.34/5.93	1.89 ± 0.13	0.63 ± 0.38	0.33^*^ ± 0.16	1.09 ± 0.19
Chaperones										
68	gi|460389504	Protein disulfide-isomerase-like, *Solanum lycopersicum*	82	6	2	55.11/4.81	0.53 ± 0.02	0.55 ± 0.01	3.51^*^ ± 0.28	3.39^*^ ± 0.22
69	gi|460395973	Heat shock protein 83, *Solanum lycopersicum*	76	1	1	90.77/5.23	0.96 ± 0.04	1.99 ± 0.03	19.73^*^ ± 0.24	9.53^*^ ± 0.23
70	gi|460395973	Heat shock protein 83, *Solanum lycopersicum*	193	3	3	90.77/5.23	1.24 ± 0.23	3.10^*^ ± 0.37	2.07^*^ ± 0.26	0.82 ± 0.07
71	gi|762844	Hsc70, *Solanum lycopersicum*	115	4	2	71.87/5.18	5.38^*^ ± 0.40	0.60 ± 0.15	1.04 ± 0.28	9.20^*^ ± 0.21
72	gi|170386	Glucose-regulated protein 78, *Solanum lycopersicum*	211	12	3	41.32/8.51	2.45^*^ ± 0.40	0.73 ± 0.14	1.31 ± 0.26	4.32^*^ ± 0.41
73	gi|145341034	AAA-metalloprotease FtsH, *Ostreococcus lucimarinus*	131	6	4	67.77/5.23	0.92 ± 0.05	0.63 ± 0.29	0.44^*^ ± 0.01	0.75 ± 0.32
Signal transduction										
74	gi|1168191	14-3-3 protein 4 OS, *Solanum lycopersicum*	58	25	4	29.40/4.69	0.78 ± 0.33	0.67 ± 0.29	0.10^*^ ± 0.04	0.11^*^ ± 0.05
75	gi|3041662	14-3-3 protein 3, *Solanum lycopersicum*	165	12	2	29.40/4.74	0.56 ± 0.23	1.33 ± 0.24	0.21^*^ ± 0.04	0.08^*^ ± 0.02
76	gi|460405902	Plasma membrane-associated cation-binding protein 1, *Solanum lycopersicum*	95	6	1	21.98/5.03	1.71 ± 0.37	0.99 ± 0.19	0.20^*^ ± 0.04	0.34^*^ ± 0.04
77	gi|590715109	Transducin family protein/WD-40 repeat family protein isoform 1, *Theobroma cacao*	57	2	1	87.16/6.40	0.82 ± 0.01	0.18^*^ ± 0.01	2.87^*^ ± 0.03	12.76^*^ ± 0.11
78	gi|460393840	Proliferation-associated protein 2G4-like, *Solanum lycopersicum*	134	7	2	43.07/6.41	2.16^*^ ± 0.17	0.59 ± 0.15	0.27^*^ ± 0.09	0.97 ± 0.19
79	gi|525314284	Hop-interacting protein THI113, *Solanum lycopersicum*	117	10	2	37.34/5.82	0.93 ± 0.14	0.11^*^ ± 0.07	0.24^*^ ± 0.18	1.96 ± 0.10
Detoxification and antioxidation										
80	gi|350536897	Cytosolic ascorbate peroxidase 1, *Solanum lycopersicum*	113	14	2	27.73/5.61	1.54 ± 0.26	0.66 ± 0.06	0.42^*^ ± 0.09	0.96 ± 0.09
81	gi|350536897	Cytosolic ascorbate peroxidase 1, *Solanum lycopersicum*	115	7	1	27.73/5.61	7.20^*^ ± 0.13	7.42^*^ ± 0.32	6.03^*^ ± 0.16	5.85^*^ ± 0.07
82	gi|350539113	Ascorbate peroxidase, *Solanum lycopersicum*	141	5	2	42.38/8.65	1.90 ± 0.20	0.75 ± 0.18	0.32^*^ ± 0.04	0.81 ± 0.07
83	gi|460373807	Putative lactoylglutathione lyase, *Solanum lycopersicum*	126	15	5	32.95/5.95	0.82 ± 0.22	0.17^*^ ± 0.07	0.44^*^ ± 0.21	2.05^*^ ± 0.24
84	gi|50400860	Monodehydroascorbate reductase, *Solanum lycopersicum*	255	17	5	47.12/5.77	0.85 ± 0.07	0.49^*^ ± 0.17	0.40^*^ ± 0.18	0.69 ± 0.16
85	gi|251895	Polyphenol oxidase, *Solanum lycopersicum*	169	7	5	66.83/6.61	1.96 ± 0.06	2.87^*^ ± 0.04	15.32^*^ ± 0.10	10.49^*^ ± 0.42
86	gi|460397526	Heme-binding protein 2, *Solanum lycopersicum*	183	17	3	26.12/7.59	0.84 ± 0.08	0.82 ± 0.05	4.29^*^ ± 0.25	4.41^*^ ± 0.18

^a^Numbering corresponds to the 2-DE in Fig. 3

^b^Accession number from the NCBI nr database

^c^Names and species of the proteins obtained via the MASCOT software from the NCBI nr database

^d^Molecular weight search (MOWSE) score probability for the entire protein identified by the MASCOT software

^e^The sequence coverage of identified proteins

^f^Total numbers of identified peptides

^g^Molecular weight and isoelectric point of the identified proteins

^h^Protein abundance ratio of Treatment/Control tomato cultivars, with each value representing the mean value ± SD of three biological replicates

^∗^Indicates significant (more than 2.0-fold or less than 0.50-fold) difference between control and treatment tomato cultivars

### Functional classification of identified proteins involved in TYLCV infection

**Fig. 5:**
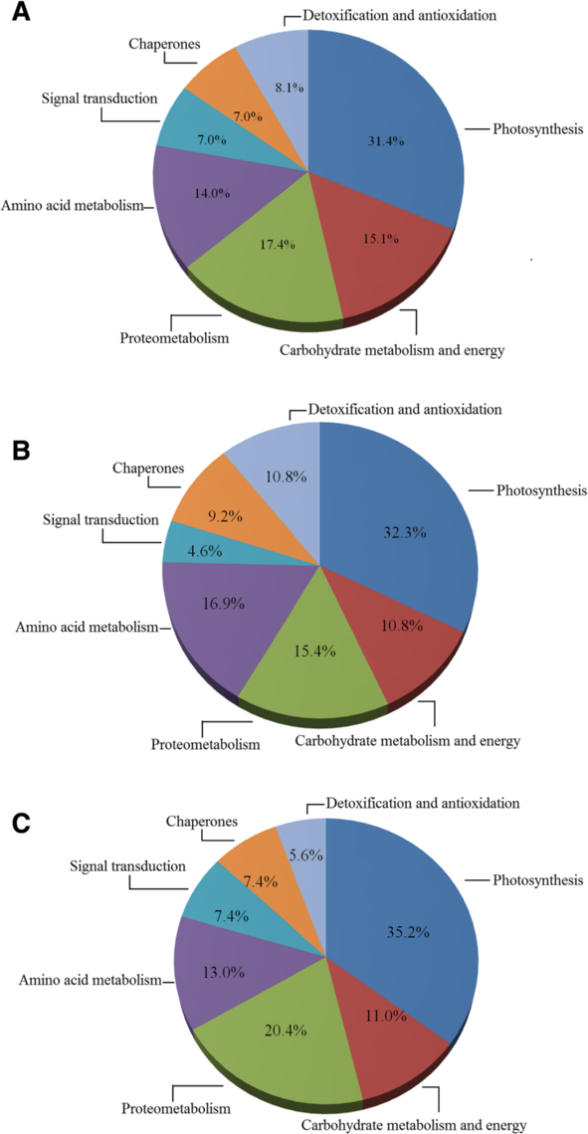
Functional classification of differentially expressed proteins in the leaves of two tomato cultivars under TYLCV infection. **a** Proportion of 86 differentially expression proteins identified in two tomato cultivars. **b** Proportion of functional classification of differentially expressed proteins in tomato genotypes ‘Zheza-301’. **c** Proportion of functional classification of differentially expressed proteins in genotypes ‘Jinpeng-1’

All 86 differentially expressed proteins were successfully identified as plant proteins. As shown in Fig. 5a, the proteins were classified into the following functional classes according to the Kyoto Encyclopedia of Genes and Genomes (KEGG) (http://www.kegg.jp/kegg/pathway.html): 27 photosynthesis (31.4 %), 15 proteometabolism (17.4 %), 13 carbohydrate metabolism and energy (15.1 %), 12 amino acid metabolism (14.0 %), 7 detoxification and antioxidation (8.1 %), 6 signal transduction (7.0 %), 6 chaperones (7.0 %). The functional classes of identified proteins in two different tomato genotypes ‘Zheza-301’ and ‘Jinpeng-1’ were the same (Fig. 5b-c).

Among the 27 proteins involved in photosynthesis, 11 enzymes including CAB (chlorophyll a-b binding-protein, spots 1–3) [23, 24], OEE1 (oxygen-evolving enhancer protein1, spots 4, 5) [25, 26] and LFNR (ferredoxin–NADP reductase, leaf-type isozyme, spots 22, 23) play important roles in light-dependent reactions (Table 1). The other 16 enzymes, such as ribulose-1,5-bisphosphate carboxylase (RuBisCO, spots 11–14) and carbonic anhydrase (spots 24, 25), participate in the Calvin cycle [27]. Thirteen proteins are involved in carbohydrate metabolism and energy, such as GAPDH (glyceraldehyde 3-phosphate dehydrogenase, spot 28), AH (aconitate hydratase, spot 33), EA (enolase, spot 35), ID (isocitrate dehydrogenase, spot 38) and CHI (chitinase, spot 40). CAR (wound-inducible carboxypeptidase precursor, spots 41, 42), CDC48 (cell division cycle protein 48 homolog, spots 51, 52), PRO (subtilisin-like protease precursor, spot 54) and eukaryotic initiation factor 4A-2 (spots 46, 47) take part in proteometabolism. Several proteins are identified as being involved in signal transduction or as chaperones, such as HSP83 (spots 69, 70), HSC70 (spot 71) and 14-3-3 protein (spots 74, 75). Detoxification and antioxidation are essential for plants to survive. When faced with TYLCV infection, PPO (polyphenol oxidase, spot 85), Glo I (lactoylglutathione lyase, spot 83) and APX (ascorbate peroxidase, spots 80–82) are detected in both tomato genotypes. Twelve proteins are identified as being related to amino acid metabolism, including GLDC (glycine dehydrogenase (decarboxylating), spots 60–62), THD (threonine deaminase, spots 63–65), MAT (S-adenosylmethionine synthase 2, spot 59) and CYS (cysteine synthase, spots 66, 67).

### Differentially expressed proteins identified in resistant and susceptible tomato cultivars after TYLCV infection

**Fig. 6:**
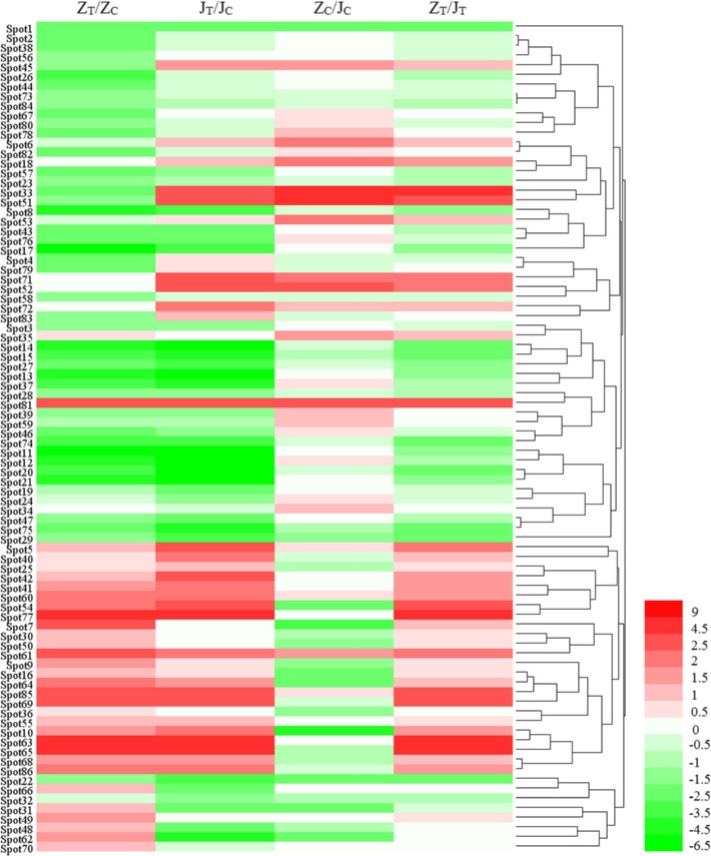
Heat map representation and hierarchical clustering of 86 identified proteins in resistant cultivar ‘Zheza-301’ and susceptible cultivar ‘Jinpeng-1’ after TYLCV infection. The protein abundance ration of each spot was normalized by the log2 transformed to represent color scores. Red represented high expression, and green represented low expression. Z represented ‘Zheza-301’, J represented ‘Jinpeng-1’, T represented ‘treatment’, C represented ‘control’

Expression patterns in terms of the protein abundance ratios of 86 identified proteins were different between the two tomato cultivars. A heat map of differentially expressed protein spots provides an overview of protein expression modification in the two TYLCV-infected tomato genotypes (Fig. 6). In general, most proteins in ‘Zheza-301’ showed down-regulated expression profiles. In ‘Jinpeng-1’, the proportions of up- and down-regulated proteins were almost the same. In ‘Zheza-301’, 71 proteins were modulated between the TYLCV-infected and control leaf samples, with the expression levels of 28 proteins increased and 43 proteins decreased. In ‘Jinpeng-1’, 59 proteins appeared to be altered, and of these 28 and 31 were up-regulated and down-regulated, respectively (Fig. 4). Among the 86 protein spots, expression levels of 17 proteins were up-regulated in both ‘Zheza-301’ and ‘Jinpeng-1’, including OEE1 (spot 5), HSP83 (spot 69) and PPO (spot 85). A total of 24 protein spots were identified with down-regulated expression levels in both cultivars, including 14-3-3 protein (spots 74, 75) and RuBisCO (spots 11–14). Some proteins showed opposite expression patterns in the two cultivars. The expression levels of spots 48, 62 and 66 showed up-regulation in ‘Zheza-301’, but down-regulation in ‘Jinpeng-1’ (Table 2). The differences in expression patterns of proteins indicate that several proteins play different roles in resistant and susceptible tomato cultivars in response to TYLCV infection.

**Table 2:** Proteins differentially expressed between resistant and susceptible tomato cultivars

^a^Spot no.	^b^Accession number	^c^Protein name	^d^Z_T_/Z_C_	^d^J_T_/J_C_
Up-regulated proteins				
Spot5	gi|460372520	Oxygen-evolving enhancer protein 1	2.21^*^ ± 0.44	10.57^*^ ± 0.52
Spot10	gi|100380	Ribulose bisphosphate carboxylase activase	3.25^*^ ± 0.44	5.57^*^ ± 0.43
Spot41	gi|350535160	Wound-inducible carboxypeptidase precursor	3.62^*^ ± 0.30	5.27^*^ ± 0.22
Spot42	gi|350535160	Wound-inducible carboxypeptidase precursor	2.72^*^ ± 0.19	6.05^*^ ± 0.30
Spot54	gi|350536267	Subtilisin-like protease precursor	4.17^*^ ± 0.29	14.46^*^ ± 0.40
Spot55	gi|460381101	28 kDa ribonucleoprotein	2.17^*^ ± 0.70	2.05^*^ ± 0.25
Spot60	gi|460399143	Glycine dehydrogenase (decarboxylating)	4.02^*^ ± 0.25	5.51^*^ ± 0.19
Spot61	gi|460399143	Glycine dehydrogenase (decarboxylating)	6.81^*^ ± 0.38	5.09^*^ ± 0.21
Spot63	gi|170458	Threonine deaminase, partial	5.90^*^ ± 0.09	7.29^*^ ± 0.29
Spot64	gi|170458	Threonine deaminase, partial	5.28^*^ ± 0.62	2.96^*^ ± 0.28
Spot65	gi|170458	Threonine deaminase, partial	4.35^*^ ± 0.03	7.62^*^ ± 0.14
Spot68	gi|460389504	Protein disulfide-isomerase-like	3.51^*^ ± 0.28	3.39^*^ ± 0.22
Spot69	gi|460395973	Heat shock protein 83	19.73^*^ ± 0.24	9.53^*^ ± 0.23
Spot77	gi|590715109	Transducin family protein / WD-40 repeat family protein isoform 1	2.87^*^ ± 0.03	12.76^*^ ± 0.11
Spot81	gi|350536897	Cytosolic ascorbate peroxidase 1	6.03^*^ ± 0.16	5.85^*^ ± 0.07
Spot85	gi|251895	Polyphenol oxidase	15.32^*^ ± 0.10	10.49^*^ ± 0.42
Spot86	gi|460397526	Heme-binding protein 2	4.29^*^ ± 0.25	4.41^*^ ± 0.18
Down-regulated proteins				
Spot1	gi|407970998	Chlorophyll a-b binding protein 4	0.22^*^ ± 0.13	0.18^*^ ± 0.09
Spot3	gi|460405507	Chlorophyll a-b binding protein 8	0.46^*^ ± 0.03	0.36^*^ ± 0.13
Spot8	gi|723739979	Ribulose bisphosphate carboxylase/oxygenase activase	0.07^*^ ± 0.05	0.16^*^ ± 0.01
Spot11	gi|488453358	Ribulose 1,5-bisphosphate carboxylase	0.04^*^ ± 0.01	0.01^*^ ± 0,01
Spot12	gi|488453358	Ribulose 1,5-bisphosphate carboxylase	0.06^*^ ± 0.01	0.02^*^ ± 0.01
Spot13	gi|488453358	Ribulose 1,5-bisphosphate carboxylase	0.05^*^ ± 0.01	0.05^*^ ± 0.01
Spot14	gi|488453358	Ribulose 1,5-bisphosphate carboxylase	0.09^*^ ± 0.01	0.04^*^ ± 0.01
Spot15	gi|1293000	Ribulose-1,5-bisphosphate carboxylase/oxygenase large subunit	0.10^*^ ± 0.05	0.06^*^ ± 0.01
Spot17	gi|21069067	Ribulose 1,5-bisphosphate carboxylase/oxygenase large subunit	0.02^*^ ± 0.01	0.09^*^ ± 0.08
Spot20	gi|92087012	Ribulose bisphosphate carboxylase large chain OS	0.09^*^ ± 0.01	0.01^*^ ± 0.01
Spot21	gi|92087012	Ribulose bisphosphate carboxylase large chain OS	0.08^*^ ± 0.01	0.01^*^ ± 0.01
Spot22	gi|460375527	Ferredoxin--NADP reductase, leaf-type isozyme	0.42^*^ ± 0.10	0.14^*^ ± 0.02
Spot27	gi|460372959	Peptidyl-prolyl cis-trans isomerase CYP38	0.26^*^ ± 0.05	0.15^*^ ± 0.04
Spot28	gi|469517896	Glyceraldehyde-3-phosphate dehydrogenase	0.46^*^ ± 0.03	0.44^*^ ± 0.13
Spot29	gi|460414390	ATP synthase delta chain	0.38^*^ ± 0.05	0.11^*^ ± 0.03
Spot37	gi|460373820	ATP synthase gamma chain	0.09^*^ ± 0.01	0.05^*^ ± 0.01
Spot39	gi|350535679	Cytosolic NADP-malic enzyme	0.48^*^ ± 0.07	0.49^*^ ± 0.20
Spot43	gi|350534564	26S protease regulatory subunit 6A homolog	0.20^*^ ± 0.07	0.30^*^ ± 0.04
Spot46	gi|460415494	Eukaryotic initiation factor 4A-2	0.35^*^ ± 0.02	0.37^*^ ± 0.02
Spot47	gi|460399092	Eukaryotic initiation factor 4A-2	0.49^*^ ± 0.22	0.22^*^ ± 0.05
Spot57	gi|460404838	Adenosylhomocysteinase	0.21^*^ ± 0.06	0.45^*^ ± 0.19
Spot74	gi|1168191.1	14-3-3 protein 4 OS	0.10^*^ ± 0.04	0.11^*^ ± 0.05
Spot75	gi|3041662	14-3-3 protein 3	0.21^*^ ± 0.04	0.08^*^ ± 0.02
Spot76	gi|460405902	Plasma membrane-associated cation-binding protein 1	0.20^*^ ± 0.04	0.34^*^ ± 0.04
Opposite expression proteins				
Spot31	gi|460397188	Thiamine thiazole synthase	2.25^*^ ± 0.07	0.23^*^ ± 0.17
Spot33	gi|723747143	Aconitate hydratase	0.26^*^ ± 0.02	5.77^*^ ± 0.85
Spot45	gi|460400419	Elongation factor G	0.47^*^ ± 0.01	3.29^*^ ± 0.39
Spot48	gi|460391817	Elongation factor TuB	2.45^*^ ± 0.23	0.34^*^ ± 0.03
Spot51	gi|460391351	Cell division cycle protein 48 homolog	0.38^*^ ± 0.04	5.90^*^ ± 0.81
Spot62	gi|460370413	Glycine dehydrogenase	3.33^*^ ± 0.16	0.07^*^ ± 0.02
Spot66	gi|460398434	Cysteine synthase	2.11^*^ ± 0.22	0.33^*^ ± 0.04
Spot83	gi|460373807	Putative lactoylglutathione lyase	0.44^*^ ± 0.21	2.05^*^ ± 0.24

^a^Numbering corresponds to the 2-DE in Fig. 3

^b^Accession number from the NCBI nr database

^c^Names of the proteins obtained via the MASCOT software from the NCBI nr database

^d^Protein abundance ratio of Treatment/Control tomato cultivars, with each value representing the mean value ± SD of three biological replicates

^∗^Indicates significant (more than 2.0-foldor less than 0.50-fold) difference between control and treatment tomato cultivars

### Expression analysis of *Ty-1* and *Ty-5* genes after TYLCV infection in tomato

**Fig. 7:**
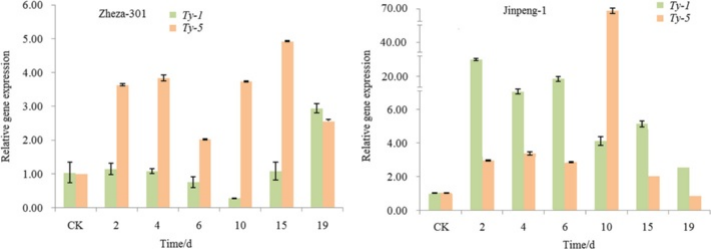
Relative expression of *Ty-1* and *Ty-5* after TYLCV infection in tomato

Two genes, namely, *Ty-1* and *Ty-5*, associated with the Ty locus, have been identified in tomatoes. The expression patterns of *Ty-1* and *Ty-5* were analyzed using qRT-PCR in ‘Zheza-301’ and ‘Jinpeng-1’ tomato cultivars (Fig. 7). In ‘Zheza-301’, the expression pattern of *Ty-5* was up-regulated at six time points after infection (2, 4, 6, 10, 15 and 19 dpi), and a peak was reached at 15 dpi with a 5-fold increase. The expression level of *Ty-1* was not noticeably increased until 19 dpi, when it increased by approximately 3-fold. The expression patterns of *Ty-1* and *Ty-5* were both up-regulated in ‘Jinpeng-1’. The expression level peaks of *Ty-1* and *Ty-5* in ‘Jinpeng-1’ were observed at 2 dpi with a 30-fold increase and at 10 dpi with a 70-fold increase, respectively.

### Expression profile analysis of proteins involved in TYLCV infection in tomato

**Fig. 8:**
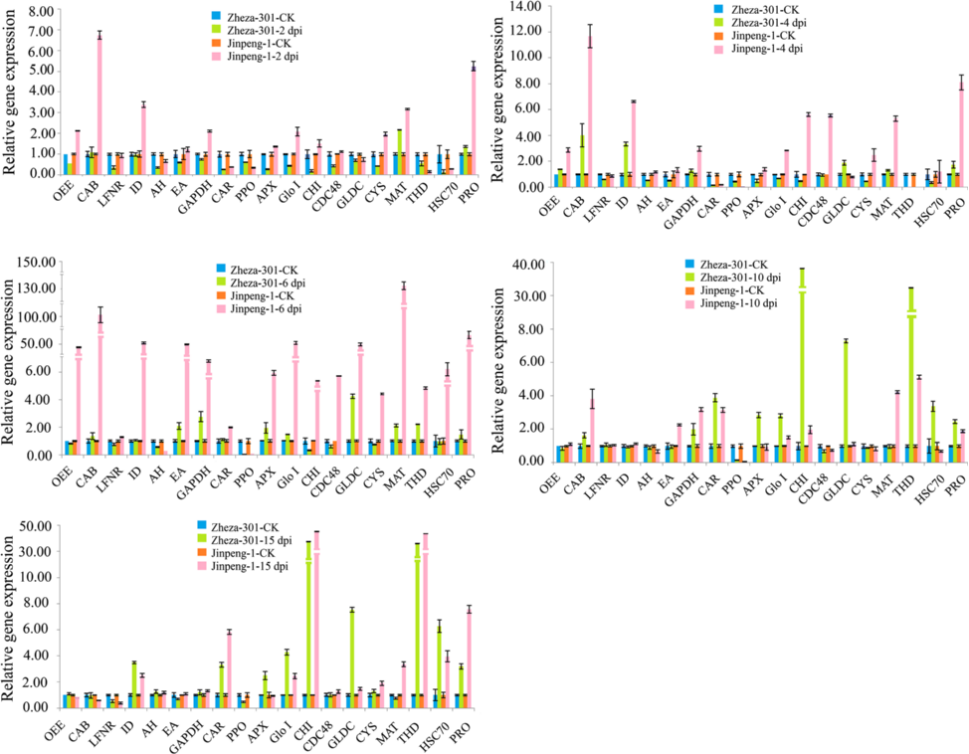
Expression patterns of 19 selected genes identified by the proteomics analysis at different TYLCV infection stages in tomato

**Fig. 9:**
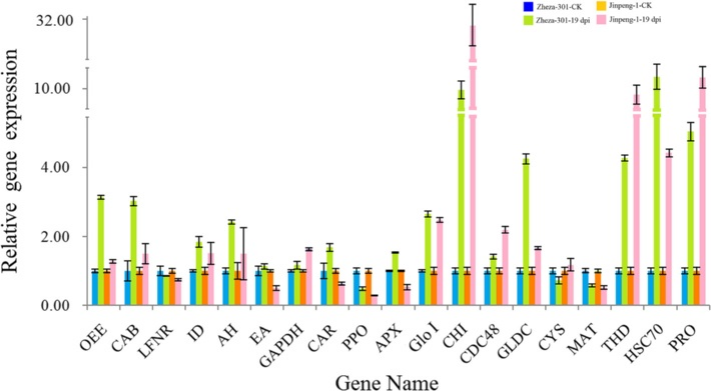
Relative expression of selected genes in response to TYLCV infection at 19 dpi in tomato

Nineteen genes encoding identified proteins, namely, *OEE*, *CAB*, *LFNR*, *ID*, *AH*, *EA*, *GAPDH*, *CAR*, *PPO*, *APX*, *Glo I*, *CHI*, *CDC48*, *GLDC*, *CYS*, *MAT*, *THD*, *HSC70* and *PRO*, were selected on the basis of our 2-DE results and subjected to expression pattern analysis. To determine where the expression of the 19 selected genes varies in different disease infection stages, qRT-PCR was conducted at six time points after infection (2, 4, 6, 10, 15 and 19 dpi) (Figs. 8 and 9).

The expression levels of *CHI*, *AH*, *HSC70* and *MAT* in early stages (2, 4 and 6 dpi) were different from those at 19 dpi. At 2, 4 and 6 dpi, *CHI*, *AH* and *HSC70* were negatively regulated in ‘Zheza-301’. Expression of *CHI*, *AH* and *HSC70* was up-regulated at 19 dpi (Figs. 8 and 9). The expression patterns of the genes that function in detoxification and antioxidation varied among the six infection periods in the two tomato cultivars. Glo I can convert 2-oxoaldehydes into less reactive 2-hydroxyacids by cooperation with Glo II. In ‘Zheza-301’, the transcription levels of *Glo I* and *APX* were decreased at 2 and 4 dpi (Fig. 8). At 19 dpi, the transcription levels of *Glo I* and *APX* in ‘Zheza-301’ were up-regulated (Fig. 9). The expression patterns of *PPO* and *PRO* were negatively and positively regulated, respectively, in both tomato cultivars at all time points after infection. Previous studies have revealed that CDC48 can impair tobacco mosaic virus (TMV) movement by removing virus-encoded MP from the endoplasmic reticulum transport pathway and by promoting the interference of MP with microtubule dynamics. The expression levels of *CDC48* in ‘Jinpeng-1’ were higher than those in ‘Zheza-301’ at all time points. At 2, 4 and 6 dpi, the expression patterns of *LFNR*, *AH*, *CHI*, *CDC48*, *CYS*, *HSC70* and *PPO* were down-regulated in ‘Zheza-301’. By contrast, the expression levels of *OEE*, *CAB*, *ID*, *AH*, *CAR*, *APX*, *Glo I*, *CHI*, *CDC48*, *GLDC*, *THD*, *HSC70* and *PRO* were up-regulated in ‘Zheza-301’ at 19 dpi. Therefore, these genes may respond to TYLCV infection via a positive regulatory mechanism (Figs. 8 and 9).

### Comparative analysis of expression patterns detected using qRT-PCR and 2-DE

The expression patterns at 19 dpi observed using qRT-PCR and 2-DE were compared. In ‘Zheza-301’, five proteins, namely, OEE, CAR, GLDC, THD and PRO were positively regulated in both qRT-PCR and 2-DE results. In ‘Jinpeng-1’, the protein abundance of OEE, AH, CHI, CDC48, THD, HSC70 and Glo I increased. The expression level of LFNR was down-regulated in both analyses. The transcription levels of the genes detected using qRT-PCR were not well correlated with the protein abundance of proteins detected using 2-DE. CAB, which functions in photosynthesis, showed opposite expression patterns in the two analyses. The protein abundance of Glo I in ‘Zheza-301’ was decreased but transcription level measured by qRT-PCR was increased approximately 3-fold at 19 dpi. In 2-DE, the protein abundance of PPO in the two tomato cultivars increased by approximately 10-fold; in contrast, its transcription levels decreased, as measured using qRT-PCR (Fig. 9, Table 1).

## Discussion

Plants use compensatory strategies to protect themselves from various aggressions by pathogens such as viruses and insects. Infestation with viruliferous whiteflies can cause extensive damage in tomatoes. The proteins identified in control and TYLCV-infected tomato cultivars were classified into different groups, such as defense-related proteins, chaperones and signal transduction proteins. These groups played different roles in response to TYLCV infection. Several of the proteins identified produced multiple spots with different *pI* and molecular masses, which indicates the presence of isoforms and posttranslational modification [28]. The ability to resist TYLCV infection varies between different tomato cultivars. Previous studies have shown that ‘Zheza-301’ and ‘Jinpeng-1’ exhibit different resistance to TYLCV infection [15, 29]. After TYLCV infection for two months, ‘Zheza-301’ had resistance ability with 45.0 % disease incidence and with 9.0 disease indexes. ‘Jinpeng-1’ showed susceptible to TYLCV infection with 90.0 % disease incidence and 60.9 disease indexes [29]. The two tomato cultivars ‘Zheza-301’and ‘Jinpeng-1’ were thus chosen to further analyze the mechanism of the response to TYLCV infection.

### Responses to TYLCV infection in resistant and susceptible tomato cultivars at different stages

Responses to TYLCV infection in tomatoes involve a complicated defense mechanism that is related to transcription factors and gene regulatory networks involving mitogen-activated protein kinases [15, 16]. Such responses are correlated with anatomical structure [30], tomato genotypes [31–33] and different infection stages [17, 33]. Glick and his colleagues reported that symptoms and responses differ between the early (before 2 weeks) and later (after 4 weeks) stages of TYLCV infection in tomatoes [34]. Tomato plants appear normal after TYLCV infection for 2 weeks, then the leaves of susceptible tomato cultivars become yellow and curly over time, and newly formed leaves are smaller and shriveled. In later stages, whole plants stop growing and this leads to severe yield loss [15, 34]. The response of tomatoes to TYLCV infection should be analyzed at different stages of infection. To determine the genes encoding resistance to TYLCV infection, some research has been conducted at early and later stages of TYLCV infection [16, 17, 33, 35, 36]. A comparative transcriptomic analysis at 3, 5 and 7 dpi has been carried out for resistant and susceptible tomato cultivars [16]. Comparative metabolomics and transcriptomics in response to TYLCV infection at 1, 3, 7 and 14 dpi have also been carried out in two tomato lines [17, 36]. Adi et al. analyzed stress responses to TYLCV infection in tomato after 4 weeks, which was sufficient to allow the development of differential coat protein (CP) aggregation [33]. Miozzi et al. investigated the transcriptional changes induced by TYLCSV infection after 6 weeks post-infection with typical systemic symptoms [35]. However, responses of tomatoes to TYLCV infection in the middle stage (~3 weeks) have not been described before now.

The responses of ‘Zheza-301’ (resistant tomato cultivar) and ‘Jinpeng-1’ (susceptible tomato cultivar) to TYLCV infection are complex and long term. A proteomics approach was used to investigate resistant and susceptible tomato cultivars and to understand the response to TYLCV infection in tomato cultivars in the middle stage (~3 weeks). At 19 dpi, a typical TYLCV phenotype was found in ‘Jinpeng-1’, but no symptoms were observed in ‘Zheza-301’. When stress conditions are encountered, plants can trigger a network of events linked to energy metabolism [37]. Most proteins in this network are involved in photosynthesis, carbohydrate metabolism and energy metabolism. This finding indicates the importance of energy metabolism in response to TYLCV infection. Changes in carbohydrate metabolism are related to energy allocation and long-term defense processes [38]. Upon TYLCV infection, changes in energy metabolism and wound response in tomato plants happen over the whole infection period [2, 7–10]. The expression patterns of 19 selected genes that encoded proteins with different functions were analyzed through qRT-PCR after 2, 4, 6, 10, 15 and 19 dpi. Changes in metabolic pathways and different expression levels were revealed through qRT-PCR and 2-DE. The symptoms in the two tomato cultivars were the combined response results caused by TYLCV infection at 19 dpi.

### Defense-related proteins

Recent studies in certain processes of virus–host interactions have reported that some oxidative stress is produced, and base damage is caused by reactive oxygen species (ROS) such as H_2_O_2_ [39–42]. Superoxide dismutase and the ascorbate-glutathione cycle are involved in the breakdown of H_2_O_2_ into H_2_O and O_2_ [43, 44]. Three isoforms of APX (spots 80–82) were identified as having different expression patterns. Spot 81 showed up-regulated expression in terms of protein abundance ratio, which was higher in ‘Zheza-301’ (Table 1). The expression level of *APX* as measured using qRT-PCR was up-regulated at 19 dpi, which was consistent with the result of 2-DE. PPOs are ubiquitous in plants and play important roles in plant defense against pests and pathogens [45–47]. PPOs can catalyze the oxygen-dependent oxidation of phenols to quinones, which may have direct antibiotic and cytotoxic activities to pathogens [45]. In our study, one PPO protein was highly expressed in both cultivars as measured using 2-DE, especially in ‘Zheza-301’, in which it increased 15-fold in protein abundance ratio. By contrast, the expression pattern of *PPO* gene measured using qRT-PCR exhibited a down-regulated expression profile both in ‘Zheza-310’ and ‘Jinpeng-1’.

In addition to PPO, APX, a lactoylglutathione lyase protein (spot 83), known as glyoxalase I (Glo I) was induced after TYLCV infection in both tomato cultivars. This enzyme catalyzes the formation of S-D-lactoylglutathione from methylglyoxal and glutathione to reduce damage [48]. Methylglyoxal is a cytotoxic and mutagenic α-ketoaldehyde that is significantly increased (2–6 fold) under abiotic stress in plants [49]. This compound can form adducts with proteins and nucleic acids, and damages cellular functions [49–52]. *Glo I*, encoding the Glo I protein, showed up-regulated transcription levels of about 3.0-fold and 2.5-fold in ‘Zheza-301’ and ‘Jinpeng-1’ at 19 dpi, respectively, while the protein abundance ratio was down-regulated in ‘Zheza-301’ (Fig. 9, Table 1). CHI is a kind of pathogenesis-related protein that can be dramatically induced by fungal, bacterial or viral attacks, and which can hydrolyze chitin in the fungal cell wall to protect the plant from biotic and abiotic stresses [52–54]. After TYLCV infection, the activity of CHI protein is increased more than in uninfected tomatoes sprayed with eugenol. In the study, CHI protein was induced in both ‘Zheza-301’ and ‘Jinpeng-1’. The protein abundance ratio in ‘Jinpeng-1’ was more than 4.2-fold that in ‘Zheza-301’. Quantitative PCR showed significantly increased expression of the *CHI* gene in ‘Zheza-301’ and ‘Jinpeng-1’ from 10 dpi and 12-fold and 35-fold increases, respectively, at 19 dpi (Figs. 8 and 9). The similar expression patterns of CHI protein in the two cultivars indicates that CHI protein plays an important role in the resistance of plants to TYLCV.

### Chaperones

Plant cells can be injured by protein misfolding or unfolding when they are confronted with adverse conditions [55, 56]. Many studies have shown that heat shock proteins (HSPs) can refold proteins to maintain cellular homeostasis and reestablish normal protein conformation under adverse conditions [24]. HSC70s are a major family of chaperones that play essential roles in a range of protein-folding processes, including protein import and translocation, and that facilitate degradation of unstable proteins [57, 58]. Another study indicated that HSP83 proteins are involved in a general regulation mechanism and control a variety of cellular functions [59, 60]. In avocados, a small 17.3 kDa heat-shock protein is induced after infection with the oomycete *Phytophthora cinnamomi* [18]. *HSP70* is over-represented in inoculated cashew plants after *Lasiodiplodia theobromae* infection [19]. In this study, HSC70 and two isoforms of HSP83 were identified in both ‘Zheza-301’ and ‘Jinpeng-1’ with up-regulated protein abundance ratios after TYLCV infection. HSP83 and HSC70 showed higher protein abundance ratios, which increased 9.5-fold and 9.2-fold, respectively, in‘Jinpeng-1’. As shown in Fig. 9, the transcription levels of *HSC70* were up-regulated in both tomato cultivars with 12-fold (in ‘Zheza-301’) and 8-fold (in ‘Jinpeng-1’) higher expression. All these results indicate that HSPs play a crucial role in recognizing and correcting misfolded proteins under TYLCV infection in tomato.

### Signal transduction proteins

14-3-3 proteins, as important regulators of cellular signal transduction and primary metabolism in plants, are ubiquitous in eukaryotes; these proteins regulate plant development and protect plants from adverse conditions [61, 62]. Previous studies have shown that 14-3-3 protein can respond to various abiotic stresses, such as environmental stress [63], metabolism/nutrient stress [64], herbivory and wound stress [65], and biotic stress, such as *Magnaporthe grisea* and *Xanthomonas oryzae* in rice [61]. Moreover, 14-3-3 protein may control the activities of kinases and phosphatases by taking part in various signal transduction processes [66]. After TYLCV infection, the protein abundance ratios of 14-3-3 protein in ‘Zheza-301’ and ‘Jinpeng-1’ showed a significant decrease. This decrease indicates that 14-3-3 is down-regulated in response to TYLCV infection.

### Other important proteins involved in TYLCV infection

Serine carboxypeptidases play important roles in plant growth and development, being involved in protein hydrolyzation, signal transduction and response to trauma and adverse conditions [67–71]. Two isoforms of CAR (spots 41, 42) were identified as having positive protein abundance ratios in the two tomato cultivars (Table 1). CDC48 was up-regulated in both ‘Jinpeng-1’ and ‘Zheza-301’ as measured by both qRT-PCR and 2-DE (Fig. 9, Table 1). As shown in Fig. 9, the expression patterns of three genes (*OEE1*, *CAB* and *LFNR*), that encode photosynthesis-related proteins, and four energy genes (*GAPDH*, *EA*, *AH* and *ID*), related to carbon metabolism, were identified by qRT-PCR at 19 dpi. The transcription levels of *OEE1*, *CAB*, *ID* and *AH* were higher both in ‘Zheza-301’ and ‘Jinpeng-1’. *LFNR* and *EA*, encoding ferredoxin-NADP reductase and enolase, were down-regulated. *GLDC*, *CYS*, *THD* and *MAT*, which are involved in amino acid metabolism, showed different transcription profiles in the two tomato cultivars. *THD* increased 5- and 10-fold in ‘Zheza-301’ and ‘Jinpeng-1’, respectively. *MAT* was down-regulated in both tomato cultivars. The expression levels of ID were opposite in 2-DE and qRT-PCR analyses, with 2-DE showing down-regulation and qRT-PCR showing up-regulation (Fig. 9, Table 1). A complex process from the gene to the corresponding protein involves complicated translation and modification, which may cause the difference in expression levels observed between the gene and protein.

### TYLCV CP aggregation in different tomato cultivars

The CP of TYLCV forms a capsid and exhibits various functions [72]. Gorovits et al. detected TYLCV CP with weak expression levels in susceptible ‘906-4’ tomato plants at 14 dpi but not in resistant ‘902’ tomato plants until 21 dpi [31]. In our study, TYLCV CP was not detected by 2-DE in the resistant cultivar ‘Zheza-301’ or the susceptible cultivar ‘Jinpeng-1’ at 19 dpi. A total of 500 protein spots with different protein abundances were identified using PDQuest software with an automation mode (data not shown). Only 86 protein spots with obviously different expression levels were manually selected, excised and analyzed through matrix-assisted laser desorption/ionization time-of-flight mass spectrometry (MALDI-TOF-TOF MS). The CP protein spot may not have been excised; consequently, the CP protein was not detected. The CP protein may be among the 414 spots that were not analyzed.

Viral replication, transcription, translation and movement, as well as virus inoculum dose and inoculation methods, influence the aggregation level of TYLCV CP in tomato plants [31]. Different tomato genotypes and resistance to TYLCV infection also affect TYLCV CP expression. After TYLCV infection, host compounds such as chaperones, proteases and stress-induced proteins also contribute to the maintenance of small aggregates in tomato plants [32]. Genetic backgrounds and resistance to TYLCV infection differ between ‘Jinpeng-1’ and ‘906-4’. Thus, these parameters influenced the accumulation of TYLCV CP [20, 29]. To analyze TYLCV CP accumulation in tomato, we will extend the time of infection with TYLCV or use isobaric tags for relative and absolute quantification (iTRAQ) in further studies [73].

### A putative TYLCV infection response network in tomato leaves

Based on proteomic analysis, 86 proteins were identified in tomato leaves at 19 dpi. A TYLCV infection response network was proposed that included the majority of the 86 TYLCV-responsive proteins, which are involved in several functional processes, such as systemic acquired resistance, redox homeostasis, photosynthesis rate, energy metabolism, proteolysis and amino acid hydrolysis.

**Fig. 10:**
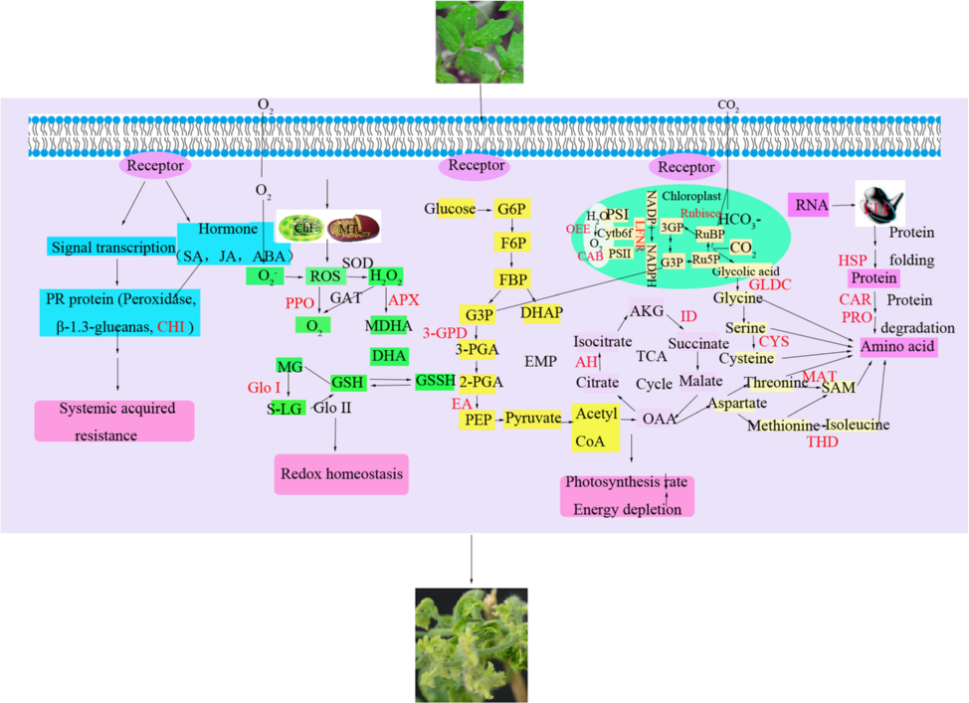
Possible TYLCV infection response network in tomato leaves. Red color represents significantly differentially expressed proteins identified in the study

As shown in Fig. 10, tomato leaves perceive a stress signal after TYLCV infection, through some receptors located on the membrane and transmit it to cellular mechanisms by signal transduction processes that alter many cellular and metabolic processes. The level of certain hormones such as salicylic acid (SA), jasmonicacid (JA), abscisic acid (ABA) and some pathogenesis-related (PR) proteins including CHI and β-1.3-glucanase were induced in response to TYLCV infection and confer systemic acquired resistance [32, 74]. The increase in ROS content after TYLCV infection could cause an imbalance of redox in tomato leaves. To maintain redox homeostasis, the synthesis of antioxidation enzymes such as SOD, APX, Glo I and PPO, increases to decrease the levels of ROS and allow the cell to achieve redox balance. The photosynthetic rate of tomato leaves is impaired by the down-regulation of RuBisCO, LFNR and CAR protein (Table 1). Energy metabolism of tomatoes under TYLCV infection is increased by up-regulation of *ID* and *AH*, which improves the ability of tomato leaves to respond to TYLCV infection and decrease the damage (Fig. 9). After TYLCV infection, protein biosynthesis of tomato leaves is inhibited through the down-regulation of EF-4A-2, whereas proteolysis increases because of up-regulation of PRO and CAR proteins. Amino acid metabolism of tomato leaves is enhanced by the over-expression of THD and GLDC proteins.

Through the changes of cellular signal transduction and metabolic processes, tomato leaves strive to resist TYLCV infection and adapt to or decrease the damage caused by TYLCV. In the current study, a putative interaction network between tomato leaves and TYLCV infection provides us with important information about the strategy of cellular activities responding to TYLCV infection in tomato leaves.

### Comparative analysis with other ‘omics’ used to study plant–virus interactions

‘Omics’ technologies, including transcriptomics, proteomics and metabolomics can be applied to monitor and capture complete changes that occur under adverse conditions at transcript, protein and metabolic levels [75, 76]. In plants, geminivirus infections, including TYLCV, have rarely been subjected to transcriptomic analysis. A total of 3604 genes and 34,831 transcripts were identified through transcriptomics, as conducted by Miozzi et al. and Chen et al., respectively [16, 35]. Only 86 significantly differentially expressed proteins were identified in this study using proteomics. Changes in gene expression at the transcript level are not well correlated with alterations in protein levels because of transcript instability and post-transcriptional modification. Genes encoding different proteins involved in TYLCV infection were revealed through transcriptomic and proteomic analyses; these genes included *HSP*, *CHI* and *CYS*. Gene ontology analysis also demonstrated that genes identified through transcriptomic and proteomic analyses were mainly involved in response to biotic stress, amino acid metabolism and protein degradation. Regulatory factors, such as MYB, NAC and WRKY, could be identified through transcriptomics but not through proteomic analysis. These proteins are often difficult to investigate because of their low abundance [16, 35].

In the innate immune response of plants, small molecules can be analyzed in detail and compounds can be identified through metabolomic approaches [77]. The combination of global gene expression and metabolic profiling is a powerful approach that can provide comprehensive insights into cellular dynamics associated with pathogen infections [17, 33]. After TYLCV infection occurs, numerous metabolites are involved in several metabolic pathways, such as phenylpropanoids and lignins; these metabolites exhibit different regulatory mechanisms between resistant and susceptible tomato cultivars [17]. Glucosamine is a metabolic compound involved in ROS metabolism; the amounts of glucosamine decreased in the early stages of infection but sharply increased at 25–28 dpi [33]. The protein abundance ratio of APX (spot 81) involved in antioxidation was up-regulated in the two tomato cultivars (Table 1). The findings indicate that changes in primary and secondary metabolites occur in the defense against TYLCV infection in tomato.

Proteomics has been used to investigate the potential functional mechanisms involved in resistance to pathogen infections in tomatoes [78, 79]. Proteins, including APX and OEE, have been identified in proteomics data from TYLCV-infected, TMV-infected, and cucumber mosaic virus (CMV)-infected tomato plants [78, 80]. CHI and LAP have been identified in TYLCV-infected and TMV-infected tomato plants. After TYLCV and CMV infection occurs, photosynthesis, energy metabolism and carbon metabolism are altered in tomato plants. Proteins involved in defense responses are also induced. HSP, which functions as a chaperone, is induced at 4 weeks post-TYLCV infection and at 19 dpi [33].

## Conclusions

A comprehensive proteomics analysis in the leaves of resistant and susceptible tomato cultivars was conducted. Eighty-six protein spots were identified with more than 2-fold or less than 0.5-fold in protein abundance ratio after TYLCV infection in two cultivars. Classified into seven functional groups, proteins identified after TYLCV infection played different roles in the process of tomato–TYLCV interaction. The interaction network between tomato leaves and TYLCV infection provided us the information about the possible activities in tomato leaf cells. The results will help to find the key proteins involved in tomato–TYLCV interaction to enhance the resistance to TYLCV and obtain protection from virus infection.

## Methods

### Plant materials and TYLCV infection

In this study, resistant tomato cultivar ‘Zheza-301’, derived from T5678161-1-1-2-2 and T07-018, and susceptible tomato cultivar ‘Jinpeng-1’, a hybrid of Holland tomato cultivar 99-13A and 9708B from America, were chosen as the source for the comparative proteomic analysis. ‘Zheza-301’ was identified as carrying the Ty-2 locus, which because T07-018 was selected from the 4th generation of the ‘CLN2498E’, breeding to contain the *Ty-2* resistant gene [81]. Seeds of tomato cultivars ‘Zheza-301’ and ‘Jinpeng-1’ were obtained from the Institute of Vegetables, Zhejiang Academy of Agricultural Sciences, and Xi’an Jinpeng Seed Co., Ltd., respectively. Tomato plantlets of ‘Zheza-301’ and ‘Jinpeng-1’ were grown in a chamber under 25 °C/18 °C, 12 h/day and a relative humidity of 60–70 % [29]. Viruliferous whiteflies, provided by Provincial Key Laboratory of Agrobiology, Jiangsu Academy of Agricultural Sciences (Nanjing, China), were allowed to feed on tomato plants in an insect-proof greenhouse. Tomato plantlets at the two-leaf stage were transmitted to the insect-proof greenhouse to expose them to viruliferous whiteflies. Control plants were transmitted to an insect-proof greenhouse without viruliferous whiteflies. The leaves of TYLCV-infected tomato plants were harvested at 2, 4, 6, 10, 15 and 19 dpi. To guarantee a better comparative understanding of the development of infection between resistant and susceptible tomato cultivars, leaves of the two tomato cultivars were processed at 19 dpi; at this point, systemic symptoms, including curly yellow leaves, were apparent in ‘Jinpeng-1’ but not in ‘Zheza-301’. The leaves of control and infected tomato cultivars at 19 dpi were collected and frozen for protein extraction.

### Protein extraction and 2-DE

Three biological repeats of each sample were collected to improve accuracy. According to the Bio-Rad (Hercules, CA, USA) 2-D manual, acetone/trichloroacetic acid (TCA) precipitation method was used to extract protein with slight modifications. Tomato leaf sample powder was briefly suspended in 10 % w/v TCA/acetone containing 1 mM phenylmethanesulfonyl fluoride (PMSF) and 0.07 % w/v β-mercaptoethanol, and held at −20 °C for 1 h. Approximately 800 μL of lysis solution (containing 7 M urea, 2 M thiourea, 4 % (w/v) 3-[(3-cholamidopropyl) dimethylammonium]-1-propanesulfonate, 65 mM dithiothreitol, 1 mM PMSF and 0.5 % v/v biolytes) was used to dissolve the vacuum-dried pellets after centrifugation and rinse. After centrifugation, the insoluble materials were removed. The Bradford method was performed to quantify the protein concentration for each sample [82]. About 1500 μg of protein of each sample was loaded on a 24 cm nonlinear gradient immobilized pH gradient strip (pH 4–7) and subjected to IEF at 20 °C: 50 v for 13 h, 100 v for 1 h, 200 v for 1 h, 1000 v for 1 h, 8000 v for 3 h, and 8000 v for a total of 110,000 VH. Afterward, 12 % sodium dodecyl sulfate polyacrylamide gel electrophoresis (SDS-PAGE) was used for the second electrophretic dimension. Proteins were observed by staining with Coomassie Brilliant Blue G-250. A gel scanner (Powerlook 2100XL, UMAX) and PDQuest software package (ver 7.2.0; Bio-Rad), with automation mode, were used to digitalize and analyze the gel images, respectively [83]. Based on the total density of gels with the parameter of percentage volume, spots were detected, matched and normalized. Subsequently, the mean relative volume of each spot by three biological repeats was computed and spots with more than 2.0-fold and less than 0.5-fold of protein abundance ration was considered differentially expressed protein. The experimental design was showed in Additional file 4: Figure S3.

### In-gel digestion and MALDI-TOF-TOF MS analysis

Differentially expressed protein spots were manually excised, washed three times with Millipore pure water and destained twice with 50 mM NH_4_HCO_3_ in 50 % acetonitrile (ACN). Afterward, 10 mM dithiothreitol in 50 mM NH_4_HCO_3_ was used to reduce alkylation with 40 mM iodoacetamide in 50 mM NH_4_HCO_3_. ACN (100 %) was employed to dry twice and was digested overnight at 37 °C with sequencing grade modified trypsin (Promega, Madison, WI, USA) in 50 mM NH_4_HCO_3_. The peptides were extracted twice with 0.1 % trifluoroacetic acid (TFA) in 50 % ACN. After being pooled and lyophilized, the resulting lyophilized tryptic peptides were dissolved in 5 mg/mL CHCA containing 0.1 % TFA and 50 % acetonitrile. A 4800 plus MALDI-TOF-TOF TM analyzer (Applied Biosystems, Foster City, CA, USA) was used for analyses.

MASCOT program online was employed to search the database of all protein spectra (http://www.matrixscience.com), against NCBInr databases. The search parameters were as follows: 0.15 Da mass tolerance for peptides and 0.25 Da mass tolerance of TOF–TOF fragments, one allowed trypsin miscleavage, carbamidomethyl of cystine (Cys) as fixed modification, oxidation of methionine (Met), and pyro-glutamicacid (Glu) formation of N-terminal glutarnine (Gln) and Glu as variable modification. To select the significant hits, the protein abundance ratio of each spot (treatment/control) with more than 2.0-fold or less than 0.5-fold was formulated as the selection criteria. Only significant hits, which were identified by the MASCOT probability analysis, were accepted.

## Bioinformatic analysis

The protein abundance ratio of each spot shown in Table 1 was analyzed and normalized using a log2 transform, and then a heat map with hierarchical clustering was produced using HemI 1.0 software [84].

### Semi-quantitative PCR and qRT-PCR analysis

The total RNA of tomato leaves was extracted using an RNA kit (RNA simple total RNA kit, Tiangen, Beijing, China) and then transcribed into cDNA using a Primer Script RT reagent kit (TaKaRa, Dalian, China), respectively. qRT-PCR with SYBR Premix *Ex Taq* was conducted using an ABI7500 (Applied Biosystems, Foster, CA, USA) according to the following procedure: 95 °C for 30 s, followed by 40 cycles at 95 °C for 5 s and 60 °C for 30 s, and melting curve analysis (61 cycles) at 65 °C for 10 s. Primer Premier 5.0 software was used to design the primer of each selected protein and *A-Tubulin* (Solyc04g077020.2) was used to regulate the expression level [85]. The expression patterns of two genes (*Ty-1* and *Ty-5*) associated with the Ty locus were assessed after TYLCV infection in the two tomato cultivars, 18S ribosomal RNA was used as reference with the following primers 5′-GCGACGCATCATTCAAATTTC-3′ and 5′-TCCGGAATCGAACCCTAATTC-3′ [22].

To detect whether there was accumulation of TYLCV DNA after TYLCV infection, semi-quantitative PCR was conducted using the primers TYLCV-01 F/R. *A-Tubulin* was used as internal control for semi-quantitative PCR and qRT-PCR. Total DNA was extracted from leaves of two tomato cultivars (control and treatment) using a DNA kit (DNAsecure Plant Kit, Tiangen, Beijing, China). The condition and parameters of PCR were 95 °C for 5 m, followed by different cycles (23, 25, 27 and 30) at 95 °C for 30 s, 54 °C for 1 m, 72 °C 1 m, then 72 °C 10 m. Primers used in the study were shown in Additional file 2: Table S1.

## Abbreviations

2-DE, two-dimensional gel electrophoresis; ABA, abscisic acid; ACN: acetonitrile; AD, aryl-al/cohol dehydrogenase related protein; AH, aconitate hydratase; APX, ascorbate peroxidase; CAB, chlorophyll a-b binding protein 4; CAR, wound-inducible carboxypeptidase precursor; CDC48, cell division cycle protein 48 homolog; CHI, chitinase; CMV, cucumber mosaic virus; CP, coat protein; CYS, cysteine synthase; dpi, day post infection; EA, enolase; GAPDH, glyceraldehyde-3-phosphate dehydrogenase; GLDC, glycine dehydrogenase (decarboxylating); Glo I, lactoylglutathione lyase; Glu, glutamicacid; ID, isocitrate dehydrogenase; iTRAQ, isobaric tags for relative and absolute quantification; JA, jasmonicacid; KEGG, kyoto encyclopedia of genes and genomes; LAP, leucine aminopeptidase; LFNR, ferredoxin--NADP reductase, leaf-type isozyme; MALDI-TOF-TOF MS, matrix-assisted laser desorption/ionization time-of-flight mass spectrometry; MAT, S-adenosylmethionine synthase 2; MG, methylglyoxal; MP, movement proteins; OEE, oxygen-evolving enhancer protein; ORF, open reading frame; *pI*, isoelectric point; PMSF, phenylmethanesulfonyl fluoride; PPO, polyphenol oxidase; PR, pathogenesis-related proteins; PRO, subtilisin-like protease precursor; qRT-PCR, quantitative real-time polymerase chain reaction; ROS, reactive oxygen species; RuBisCO: ribulose-1, 5-bisphosphate carboxylase; SA, salicylic acid; SDS-PAGE: sodium dodecyl sulfate polyacrylamide gel electrophoresis; ssDNA, single-stranded DNA; TCA, trichloroacetic acid; TFA: trifluoroacetic acid; THD, threonine deaminase; TMV, tobacco mosaic virus; TYLCV, tomato yellow leaf curly virus

## Additional files

Additional file 1: Figure S1. Symptoms of two tomato cultivars after TYLCV infection at different time. (TIF 4475 kb) Additional file 2: Table S1. Primers of the selected genes used in the text. (DOC 58 kb) Additional file 3: Figure S2. Representative protein spot maps of leaves of tomato cultivars ‘Zheza-301’ (A) and ‘Jinpeng-1’ (B). CK stands for the control plants that tomato seedlings are grown in normal environment without TYLCV infection. Treatment means that tomato seeding is grown in normal environment with TYLCV infection. 2-DE was performed using 1500 g of protein, nonlinear 24 cm IPG strips (pH 4–7) and 12 % SDS-PAGE gels for second dimension electrophoresis. (TIF 3424 kb) Additional file 4: Figure S3. Experimental design of our research. (TIF 1401 kb)
