# Supplementary figures and images for: Comparative proteomic analysis provides novel insight into the interaction between resistant vs susceptible tomato cultivars and TYLCV infection
Source: BMC Plant Biol. 2016 Jul 19;16:162. doi: 10.1186/s12870-016-0819-z (PMC4952150; doi:10.1186/s12870-016-0819-z)

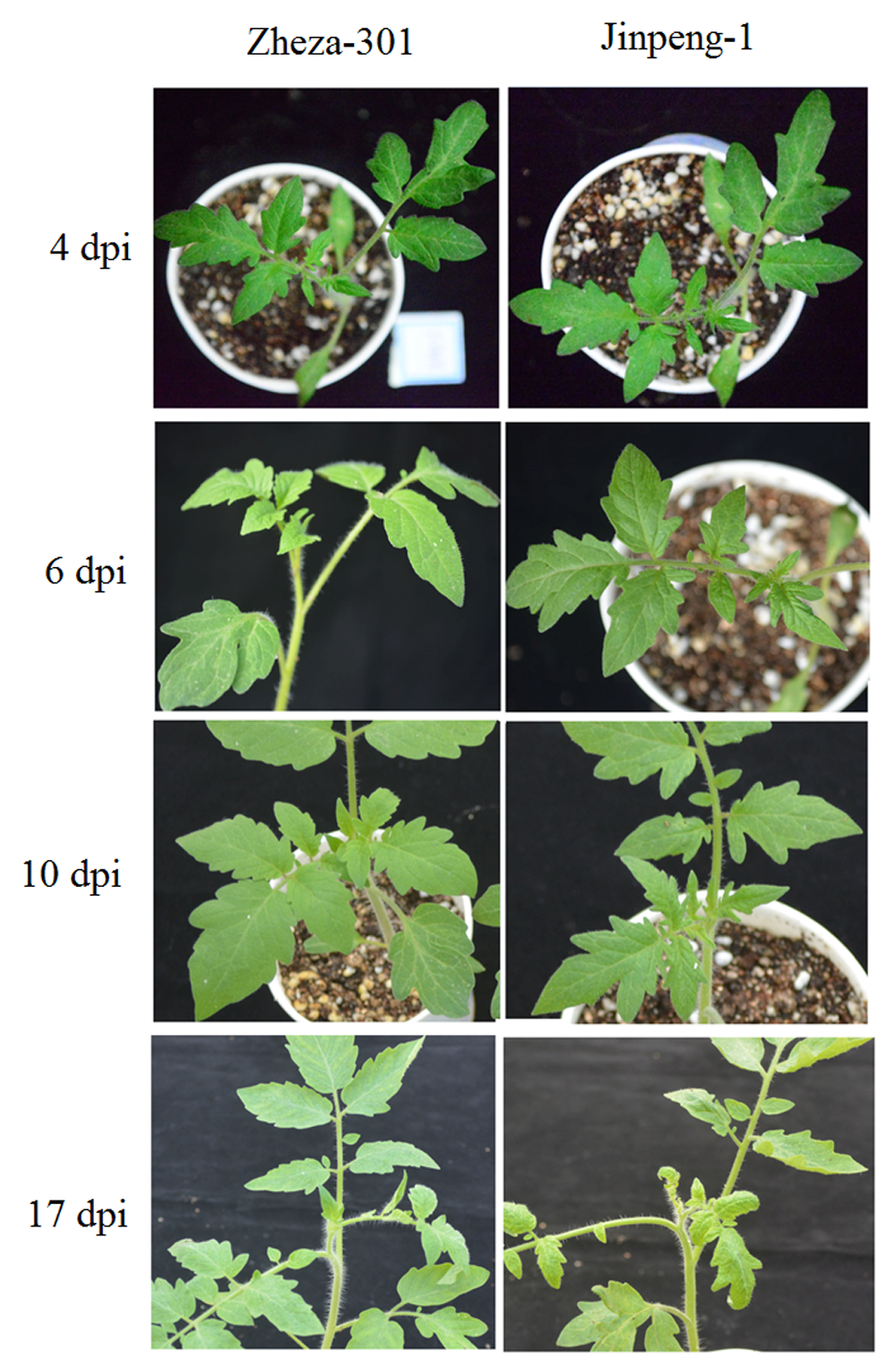

Supplement: Additional file 1: Figure S1. — Symptoms of two tomato cultivars after TYLCV infection at different time. (TIF 4475 kb) [file 12870_2016_819_MOESM1_ESM.tif]

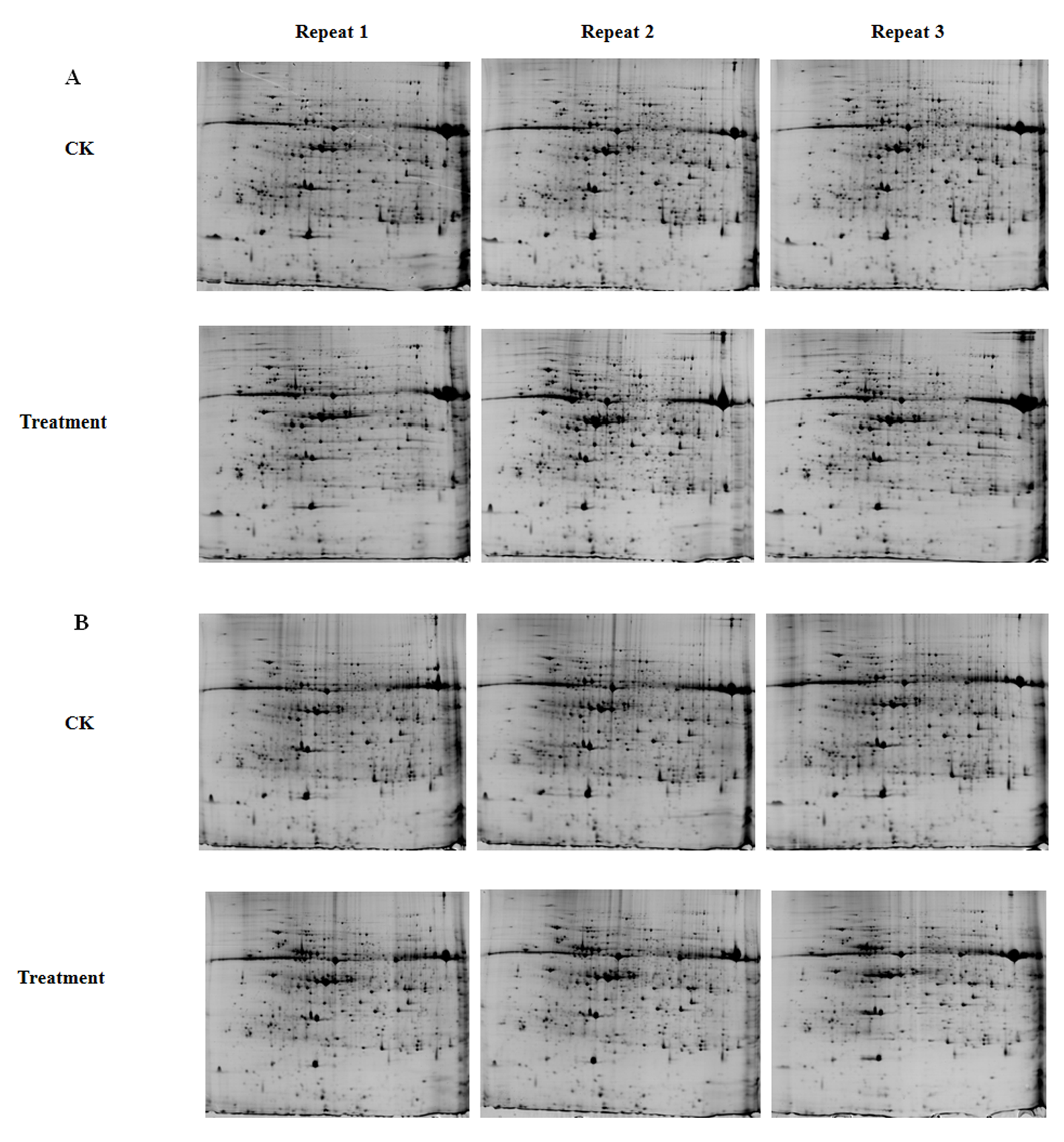

Supplement: Additional file 3: Figure S2. — Representative protein spot maps of leaves of tomato cultivars ‘Zheza-301’ (A) and ‘Jinpeng-1’ (B). CK stands for the control plants that tomato seedlings are grown in normal environment without TYLCV infection. Treatment means that tomato seeding is grown in normal environment with TYLCV infection. 2-DE was performed using 1500 g of protein, nonlinear 24 cm IPG strips (pH 4–7) and 12 % SDS-PAGE gels for second dimension electrophoresis. (TIF 3424 kb) [file 12870_2016_819_MOESM3_ESM.tif]

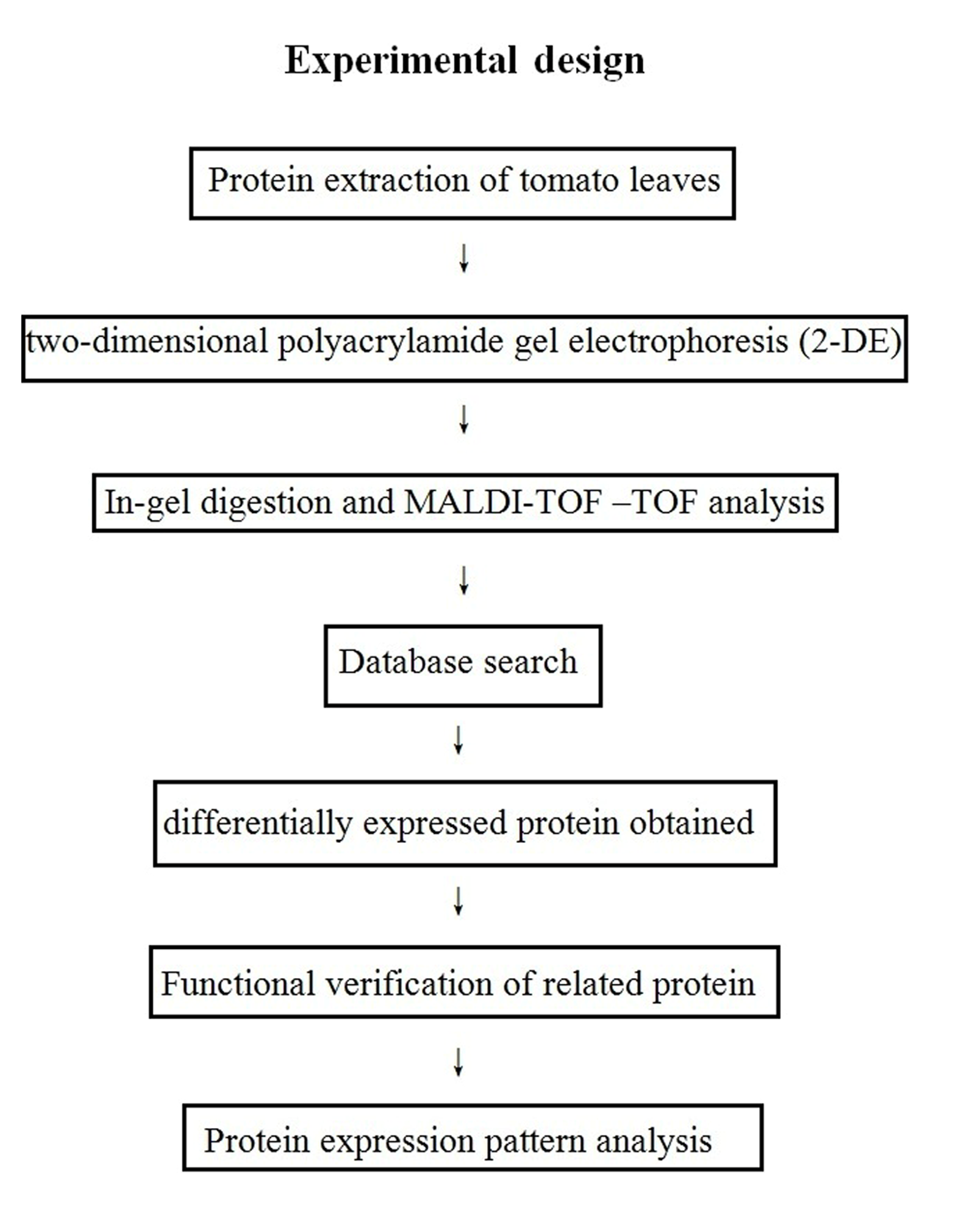

Supplement: Additional file 4: Figure S3. — Experimental design of our research. (TIF 1401 kb) [file 12870_2016_819_MOESM4_ESM.tif]
