# Supplementary material for: Comparative proteomic analysis provides novel insight into the interaction between resistant vs susceptible tomato cultivars and TYLCV infection
Source: BMC Plant Biol. 2016 Jul 19;16:162. doi: 10.1186/s12870-016-0819-z (PMC4952150; doi:10.1186/s12870-016-0819-z)
Supplement: Additional file 2: Table S1. — Primers of the selected genes used in the text. (DOC 58 kb) [file 12870_2016_819_MOESM2_ESM.doc]

| Gene | GenBank | Numbers of base | Forward primer (5'-3' ) forword | Reverse primer (5'-3' )forword |
| --- | --- | --- | --- | --- |
| *TYLCV-01* | ABG73017.1 | 777 | ATGTCGAAGCGACCAGGCGATATAAT | TTAATTTGATATTGAATCATAGAAAT |
| *CHI* | AB110610.1 | 213 | ATCACCCGTTGCACTGTCTTGTC | GCAGTATCATCACCAGCAGTACCAA |
| *Glo I* | XM_004232657.2 | 273 | TTGCCAAGTGATGCTTCGTGTG | TGGATAGCAAGATTGACAACCTTCG |
| *HSC70* | L41253.2 | 190 | TCTGCTGTTGGATGTTACACCTCTT | GTTGTTATCTCTGGTCCTGGCTCTT |
| *CDC48* | XM_004241238.2 | 151 | GACGAAGTGTGAGTGATGCCGATA | GCTCCAGCATTAGAAGTTGTGAAGG |
| *ID* | XM_004228559.2 | 181 | CCTCTGTCCTGGTGTGTCCTGAT | AGCCGTTCGTTGTTGTCCAATGT |
| *AH* | XM_004251469.2 | 225 | TGCCGAGGTTGTTCAATCAAGTGT | AAGTTCAGCAAGCAGTAAGCATCCT |
| *EA* | XM_004249525.2 | 232 | GGCAGCGTGACTGAGAGTATCG | CGGCATATACAGCATCTGAACCAAG |
| *GAPDH* | NM_001279325.2 | 284 | AAGGCTGCTGCTCACTTGAAGG | GCTCTTCCACCTCTCCAGTCCTT |
| *OEE* | XM_004232029.2 | 185 | ACATCATTCACCGTCAAGGCAGAG | TGGACAGTAACAGCAGCGTAATCAA |
| *CAB* | NM_001247810.2 | 274 | CGAGACATTTGCTAGAAACCGTGAG | GCCTCCACCAACTCTGTATCCTTC |
| *LFNR* | XM_004232447.2 | 202 | TTCCTTCATCCAAGTCCACCTCCT | TTGACAATCACACCTTCCTCCTGTT |
| *PRO* | NM_001247053.1 | 223 | GGGTGAATTGCTCGGAGGTGAAA | GTCAACTTCTGGTTCAACTCGGAGA |
| *THD* | M61914.1 | 253 | GATG5AGACCATTAGCGTTGCCGTTA | AACTCCGAGCCTATCCGAGAGC |
| *MAT* | NM_001309376.1 | 293 | TGCTAACGGTCTTGCCAGAAGG | CAGGCTTGTCCCACTTGAGAGG |
| *CYS* | XM_004247514.2 | 281 | ACAGGAGGCACAATAACAGGTTCAG | GCAGCAGCAGCACCAGATGATAT |
| *GLDC* | XM_004245053.2 | 236 | TTGATTCCTTCTCGTTGCAGCCTAA | CTCAGCAGCCTTCCTCAACTCTTC |
| *CAR* | NM_001247762.1 | 242 | AACTACCTGGCTTCAATGGCACTT | CAGAGAAGGCAGGCTACCACTTG |
| *APX* | NM_001247853.1 | 285 | TGTGCTCCTATTATGCTCCGTCTTG | TGGTGGCTCTGGCTTGTCCT |
| *PPO* | S40548.1 | 245 | CTTCTTCCTTCACCACCACCAACT | GGCAGTACCACAAGACTTGAGATCA |
| *Ty-1* | XM_010323868.1 | 162 | GGCAAAATATGCAGCCAGGCTTTCC | TCAGTATGTATACGAGGTTCGCCGT |
| *Ty-5* | KC567248.1 | 152 | CCATGAGCGTCTGGCTATTC | GGAGACATGCATTGACGAGA |
| *Tubulin* | XM_006356736.2 | 74 | TGACGAAGTCAGGACAGGAA | CTGCATCTTCTTTGCCACTG |

**Table S1 Primers of the selected genes used in the text.**
